# Nanodrug with ROS and pH Dual‐Sensitivity Ameliorates Liver Fibrosis via Multicellular Regulation

**DOI:** 10.1002/advs.201903138

**Published:** 2020-02-14

**Authors:** Liteng Lin, Hengye Gong, Rui Li, Jingjun Huang, Mingyue Cai, Tian Lan, Wensou Huang, Yongjian Guo, Zhimei Zhou, Yongcheng An, Zhiwei Chen, Licong Liang, Yong Wang, Xintao Shuai, Kangshun Zhu

**Affiliations:** ^1^ Laboratory of Interventional Radiology Department of Minimally Invasive Interventional Radiology and Department of Radiology The Second Affiliated Hospital of Guangzhou Medical University Guangzhou 510260 China; ^2^ PCFM Lab of Ministry of Education School of Material Science and Engineering Sun Yat‐Sen University Guangzhou 510275 China; ^3^ School of Pharmacy Guangdong Pharmaceutical University Guangzhou 510006 China; ^4^ College of Chemistry and Materials Science Jinan University Guangzhou 510632 China

**Keywords:** liver fibrosis, liver‐targeting delivery, microenvironment sensitive, multicellular regulation, nanodrugs

## Abstract

Liver fibrosis currently represents a global health problem without effective pharmacotherapeutic strategies. The clinical translation of polydatin, a promising natural anti‐fibrotic drug candidate with broad anti‐inflammatory and antioxidant capabilities, remains a major challenge due to its limited water solubility and tissue absorption. Herein, a polydatin‐loaded micelle (PD‐MC) based on reactive oxygen species (ROS) and pH dual‐sensitive block polymer PEG‐P(PBEM‐*co*‐DPA) is developed. The micelle exerts great potential in improving the biocompatibility of polydatin and shows highly efficient liver‐targeted drug release in response to the fibrotic microenvironment. Both in vitro and in vivo studies demonstrate that PD‐MC can significantly suppress inflammatory response and oxidative stress, reduce hepatocyte apoptosis, and avert activation of macrophages and hepatic stellate cells. More excitingly, the blank micelle itself promotes the hepatic ROS consumption at the pathologic site to provide anti‐inflammatory benefits. These favorable therapeutic virtues of targeting multiple cell types endow PD‐MC with remarkable efficacy with minimal side effects in liver fibrosis treatment. Thus, PD‐MC holds great potential to push forward the clinical application of polydatin in pharmacotherapeutic approaches against liver fibrosis.

## Graphical abstract

A reactive oxygen species and pH dual‐sensitive polydatin‐encapsulated micelle (PD‐MC) is developed for liver‐targeted drug release in response to the fibrotic microenvironment for the treatment of liver fibrosis. In vitro and in vivo results demonstrate that PD‐MC ameliorates liver fibrosis by suppressing inflammatory response and oxidative stress, reducing hepatocyte apoptosis, and averting activation of macrophages and hepatic stellate cells.

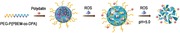

## 1 Introduction

Liver fibrosis, a reversible wound healing response that follows chronic hepatic injury, is characterized by excess accumulation of extracellular matrix (ECM).[qv: 1,2] Advanced liver fibrosis usually progresses into irreversible cirrhosis and portal hypertension, which represent the leading causes of liver‐related mortality worldwide.[qv: 3] To date, it still lacks clinically proven therapy to reverse liver fibrosis or merely halt its progression into decompensated cirrhosis and portal hypertension.[qv: 4] Especially, development of effective therapeutic agents remains an urgent challenge.

Hepatic fibrogenesis is a complex multi‐cellular pathophysiological process involving the mutual interaction between parenchymal hepatocytes and nonparenchymal liver cells including hepatic stellate cells (HSCs), Kupffer cells (KCs) and liver sinusoidal endothelial cells (LSECs).[qv: 5] Despite different etiologies such as chronic viral hepatitis, non‐alcoholic fatty liver disease, alcoholic liver disease and cholestatic liver disease, hepatocyte death acts as a triggering factor during the early stage of liver fibrosis. The destroyed hepatocytes show burst release of apoptotic cellular bodies, reactive oxygen species (ROS) and damage‐associated molecular patterns, which activate both liver resident macrophages (i.e., KCs) and HSCs.[qv: 6] The activated KCs constitute a central component of the inflammatory response in liver fibrosis via releasing vast proinflammatory and oxidation‐related mediators that irritate quiescent HSCs to differentiate into activated myofibroblasts, i.e., the principle ECM‐synthesizing cells which act as the key executor in hepatic fibrogenesis.[qv: 7] In return, the activated HSCs also promote the recruitment of macrophages from the bone marrow to augment the already‐large number of KCs, which further aggravates the deterioration of inflammation and fibrogenesis.[qv: 8] The integration of these pathological behaviors orchestrates the genesis, development and progression of liver fibrosis, in which the ongoing inflammation and ROS‐mediated oxidative stress represent two major fibrogenic factors. However, conventional therapeutic strategies to treat fibrosis mostly aim at merely reducing the activation of HSCs, leading to poor outcome due to the complex pathophysiological process. In this context, the multifunctional drugs with anti‐inflammatory and antioxidant properties to simultaneously prevent hepatocyte death and deactivate HSCs and macrophages may own better potentials to serve as therapeutic agents for liver fibrosis.

Polydatin, that is, 3,4′‐5‐trihydroxystilbene‐3‐beta‐d‐glucopyranoside, an active component originally isolated from the root of *Polygonum cuspidatum* Sieb. et Zucc. (Polygonaceae), is a traditional Chinese medicine with a long history of application in analgesic, anti‐pyretic, diuretic, and expectorant treatments.[qv: 9,10] We have recently demonstrated the hepatoprotective and antifibrotic capacities of polydatin, for example, it eliminated both inflammation and oxidative stress in a murine model of liver fibrosis.[qv: 11] Aside from the poor water solubility, the limited efficacy and safety risk of polydatin remain to be overcome for its further clinical use.[qv: 12] In recent years, targeted drug delivery systems based on microenvironment‐sensitive polymeric nanocarriers have demonstrated great potentials in increasing the bioavailability of hydrophobic therapeutic agents, improving the therapeutic efficacy and minimizing the drug side effects.[qv: 13] Hence, the development of stimuli‐responsive delivery system for polydatin which specifically release drug in pathological microenvironment of fibrotic liver to target various types of liver cells may greatly push forward the application of polydatin in liver fibrosis treatment. Considering that fibrotic liver features excessive ROS,[qv: 14] ROS‐sensitive polymeric nanocarriers for polydatin delivery may possess unique advantages. The passive entrapment of nanodrugs in the hepatic reticular endothelial system (RES)[qv: 15] promotes highly efficient delivery of polydatin to liver. Moreover, chemical reactions between the carrier and ROS may not only trigger the polydatin release but also consume ROS to reduce oxidative stress to further enhance antifibrotic therapy.[qv: 16] Therefore, a highly effective therapy and minimal side effects are possible with the polydatin‐loaded ROS‐sensitive nanodrugs.

Herein, a tailor‐made amphipathic block copolymer of polyethylene glycol (PEG) and poly(2‐((((4‐(4,4,5,5‐tetramethyl‐1,3,2‐dioxaborolan‐2‐yl)benzyl)oxy) carbonyl) oxy)ethyl methacrylate co 2‐(diisopropyl amino)ethyl methacrylate) (P(PBEM‐*co*‐DPA) was synthesized and assembled into micelle for polydatin delivery (Scheme S1, Supporting Information). The PEG corona may stabilize the micelle in aqueous media for long circulation,[qv: 17,18] and the PPBEM hydrophobic core encapsulating polydatin may react with ROS to trigger drug release and reduce the oxidative stress of liver fibrosis. In addition, the pH‐sensitive PDPA segment was introduced to endow the micelle with property of intracellularly releasing polydatin in the acidic lysosomal compartments (pH 4.5–5.5).[qv: 19] To our knowledge, this is the first example of using ROS and pH dual‐sensitive polydatin‐loaded micelle (PD‐MC) to treat liver fibrosis (Scheme S2, Supporting Information). Both in vitro and in vivo experiments were performed to explore whether PD‐MC could prevent hepatocyte death, inhibit HSC and macrophage activation, and reduce inflammation and oxidative stress to effectively ameliorate liver fibrosis.

## 2 Results

### 2.1 Polymer Synthesis and Characterization

**Figure 1:**
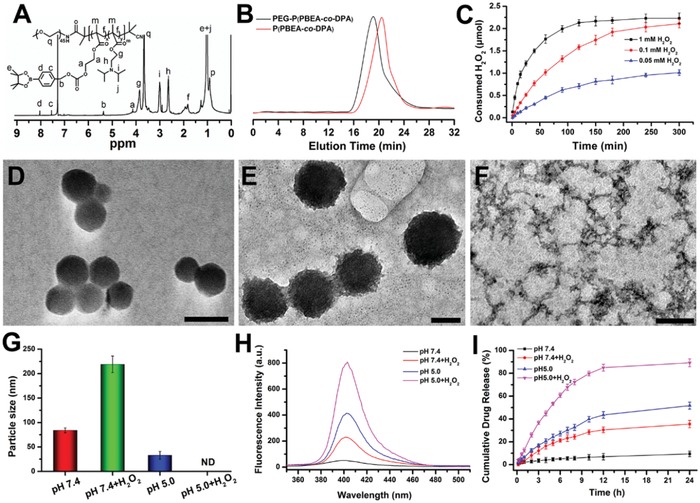
The characterization of polymer and PD‐MC. A) The ^1^H NMR spectra of the final polymer PEG‐P(PBEM‐*co*‐DPA) in CDCl_3_‐*d*. B) The GPC traces recorded for P(PBEM‐*co*‐DPA) and PEG‐P(PBEM‐*co*‐DPA). C) Consumed H_2_O_2_ after incubation of polymer (PBEM unit, 0.1 × 10^‐3^ m) in various concentrations of H_2_O_2_ solution (0.05 × 10^‐3^, 0.1 × 10^‐3^, and 1 × 10^‐3^ m) for different time points at 37 °C. The Total solution volume was 22 mL, the data were monitored by a UV–vis spectrometer at 375 nm. *n* = 3. The TEM images of D) PD‐MC in pH 7.4, E) pH 7.4 + H_2_O_2_, and F) pH 5.0 + H_2_O_2_. The scale bars represent 100 nm in (D) and (E), and 200 nm in (F), respectively. G) The particle size of PD‐MC at various conditions detected by DLS. *n* = 3. ND means none detected. H_2_O_2_ concentration if applied in (D)–(I): 0.1 × 10^‐3^ m. H) The fluorescence intensity changes of PD after incubation of PD‐MC at different conditions. I) In vitro PD release from PD‐MC solution under various conditions. *n* = 3.

The ROS and pH dual‐sensitive block copolymer PEG‐P(PBEM‐*co*‐DPA) was synthesized via multiple steps as illustrated in Scheme S1, Supporting Information. The successful synthesis of polymer was verified by ^1^H NMR and GPC analyses. In its ^1^H NMR spectrum (Figure S1, Supporting Information), 4‐(hydroxymethyl) phenylboronic acid pinacol ester (HMPBE) showed characteristic proton resonance signals at 5.24 ppm (a, —CH_2_O***H ***), 4.52 (b, —C_6_H_4_C***H***_2_OH), 7.34 ppm, and 7.64 ppm (c and d, —C_6_ ***H***_4_—). Activation of the hydroxyl group in HMPBE with CDI led to the disappearance of characteristic signal of hydroxyl group (a, —CH_2_O***H***, 5.24 ppm) but the appearance of characteristic signals of CDI group at 7.02 ppm (h, —NCH=C***H***—N=CH—), 7.63 ppm (g, —NC***H***=CH—N=CH—), and 8.32 ppm (f, —NCH=CH—N=C***H***—). The activated CDI group peak weakened along with the CDI‐HMPBE reaction with 2‐hydroxyethyl methacrylate, and the characteristic signals of 2‐hydroxyethyl methacrylate at 1.87 ppm (—CC***H***_3_=CH_2_), 4.31 ppm (—O(C***H***_2_)_2_O—), 5.69 ppm, and 6.01 ppm (—CCH_3_=C***H***_2_) validated the successful synthesis of PBEM monomer. The copolymer was synthesized through reversible addition‐fragmentation chain transfer (RAFT) copolymerization of PBEM monomer and DPA monomer using S‐1‐dodecyl‐S′‐(α,α′‐dimethyl‐α′′‐acetic acid) trithiocarbonate (DDAT) as the chain transfer agent and 2,2′‐azobis(2‐methylpropionitrile) (AIBN) as initiator. In the ^1^H NMR spectrum of P(PBEM‐*co*‐DPA)‐DDAT (Figure S2A, Supporting Information), the characteristic ^1^H NMR chemical shifts of PBEM (1.02 ppm, —OC(C***H***_3_)_2_; 3.87 ppm, —O(C***H***_2_)_2_O—; 5.20 ppm, —C_6_H_4_C***H***_2_—; 7.40 ppm, and 7.96 ppm, —C_6_ ***H***_4_—), DPA (2.65 ppm, —COOCH_2_C***H***_2_—; 3.01 ppm, —N(C***H***(CH_3_)_2_)_2_; 3.73 ppm, —COOC***H***_2_CH_2_—), and DDAT (1.02 ppm, —S(C***H***_2_)_11_CH_3_) were observed, suggesting the successful RAFT reaction. In addition, the polymerization degree of ROS‐sensitive PBEM block and pH‐sensitive DPA block in the polymeric chain was calculated to be 25 and 12, respectively, by comparing the integral intensities of the characteristic proton resonance peaks attributing to DDAT (1.02 ppm), PBEM (7.96 ppm), and DPA (2.65 ppm). In consideration of the high cytotoxicity of sulfocarbonate group, excessive AIBN was used to remove the thiolcarbonyl‐thiol end group, which was verified by the disappearance of DDAT proton resonance peaks (Figure S2B, Supporting Information). After the amidation reaction between PEG‐NH_2_ and P(PBEM‐*co*‐DPA), the characteristic resonance peak at 3.66 ppm (—OC***H***_2_C***H***_2_—) for PEG chains was clearly observed in the ^1^H NMR spectrum of PEG‐P(PBEM‐*co*‐DPA) (**Figure** **1**A). Additionally, both P(PBEM‐*co*‐DPA) and PEG‐P(PBEM‐*co*‐DPA) showed unimodal molecular weight distribution in their GPC eluograms (Figure 1B), and the final copolymer showed an obviously higher molecular weight than the prepolymer (Table S1, Supporting Information).

The PBEM pendant groups in PEG‐P(PBEM‐*co*‐DPA) contain the pinacol‐type boronic ester which could be oxidized by ROS and then hydrolyzed into quinone methide by H_2_O_2_, during which hydrophobic PBEM was converted to hydrophilic polyalcohols. The hydrolysis induced by H_2_O_2_ oxidation was confirmed by ^1^H NMR. As shown in Figure S3, Supporting Information, the characteristic proton resonance signals attributed to the phenyl protons in pinacol‐type boronic ester (7.37 and 7.83 ppm) disappeared while proton resonance signals at 6.87 and 7.25 ppm ascribed to quinone methide appeared after incubating the polymer with H_2_O_2_, which indicated the H_2_O_2_‐induced hydrolysis of the PPBEM block. The effect of H_2_O_2_ concentration on the PBEM hydrolysis of PEG‐P(PBEM‐*co*‐DPA) was studied. As shown in Figure 1C, the H_2_O_2_ consumption is within 100 min at the three different H_2_O_2_ concentrations. Moreover, higher H_2_O_2_ concentration led to faster H_2_O_2_ consumption due to quicker hydrolysis of PBEM. At 240 min, the oxidation was completed at 1 × 10^‐3^ m H_2_O_2_, and it reached 95% and 91% at 0.1 × 10^‐3^ or 0.05 × 10^‐3^ m H_2_O_2_, respectively. These results validated that the H_2_O_2_‐consuming capability of the polymer was affected by the concentration of H_2_O_2_.

### 2.2 Preparation of PD‐MC

PD‐MC was assembled from the ROS and pH dual‐sensitive block copolymer PEG‐P(PBEM‐*co*‐DPA) using sonication method. The loading content of PD in the micelle was 6.28 ± 0.12%, as determined by UV–vis spectrophotometry. The size distribution and morphology of micelle at different conditions were investigated using transmission electron microscopy (TEM) (Figure 1D–F) and dynamic light scattering (DLS) (Figure 1G). The PD‐MC showed spherical morphology with uniform particle size of 84 ± 5 nm at pH 7.4 (Figure 1D). The size was slightly bigger than that of blank micelle (71 ± 7 nm), which was likely due to the encapsulation of PD into the hydrophobic core. PD‐MC and MC both showed neutral surfaces with zeta potentials of −1.09 ± 1.15 and −2.04 ± 1.50 mV, respectively, at pH 7.4. In addition, the nanodrug showed stable particle sizes and zeta potentials demonstrating high stability in serum‐containing media (Figure S4, Supporting Information). Owing to the dual‐sensitivity of polymer, the particle size and morphology of micelle were affected by the pH and oxidizing agent. As shown in Figure 1E,G, after 0.1 × 10^‐3^ m H_2_O_2_ was added into the micelle solution, the particle size of PD‐MC significantly expanded to 219 ± 17 nm, owing to the H_2_O_2_ oxidation‐induced hydrolysis of PPBEM block which lower the hydrophobicity of PD‐MC.[qv: 20] On the contrary, following decrease of the micelle solution pH to 5.0 from 7.4, the particle size of micelle turned much smaller (35 ± 8 nm) (Figure S5, Supporting Information). Likely, the protonation of the DPA segments at pH 5.0 resulted in significantly decreased hydrophobicity of PD‐MC, which induced a disassembly of PD‐MC. However, a self‐assembly of copolymer might occur again because the unreacted PBEM segments still remained hydrophobic to drive formation of smaller micelle. Moreover, a complete collapse of micelle at pH 7.4 + 0.1 × 10^‐3^ m H_2_O_2_ was indicated by DLS and TEM analyses. These results evidenced the pH and ROS dual‐sensitivity of PD‐MC, which may facilitate the intracellular PD release.

### 2.3 pH and ROS Dual‐Sensitive PD Release of PD‐MC

Drug release under oxidative stress was evaluated in PBS containing H_2_O_2_ (0.1 × 10^‐3^ m).[qv: 21] At first, dug release was studied by measuring the fluorescence intensities of the PD‐MC solution at various conditions (Figure 1H). The PD fluorescence intensity at pH 7.4 was quite low, indicating the PD was hardly released from micelle in this condition. After adding 0.1 × 10^‐3^ m H_2_O_2_ or decreasing solution pH to 5.0 from 7.4, the PD fluorescence intensity increased obviously. However, decreasing pH appeared more effective to trigger drug release than adding H_2_O_2_. Owing to the complete disassembly confirmed in TEM and DLS analyses, the PD‐MC solution showed the highest PD fluorescence intensity at pH 5.0 + 0.1 × 10^‐3^ m H_2_O_2_. Then, the PD release from PD‐MC was quantitatively determined with high performance liquid chromatography (HPLC) analysis, which obtained results in line with the changes of fluorescence intensity at various conditions. As shown in Figure 1I, both decreasing solution pH to 5.0 and adding H_2_O_2_ facilitated the PD release, that is, 35.58% release at pH 7.4 + H_2_O_2_ and 51.70% release at pH 5.0 versus 9.38% release at pH 7.4 in 24 h. Moreover, PD release reached 89.27% in 24 h under dual stimulation (pH 5.0 + 0.1 × 10^‐3^ m H_2_O_2_), which was the complete disassembly of micelle structure. The dual‐sensitivity of PD‐MC was expected to result in quick drug release in fibrotic liver tissue with high level of ROS as and the acidic lysosomal compartments.

### 2.4 Cytotoxic Effect In Vitro

The cytotoxicities of PD and PD‐MC were evaluated by CCK‐8 assay. As shown in Figure S6, Supporting Information, LO2 cells (human hepatocytes), RAW cells (mouse macrophages), and LX‐2 cells (human HSCs) incubated with free PD exhibited almost no decrease in viabilities even at high drug concentrations up to 100 µg mL^−1^. Besides, these cell lines incubated with PD‐MC at a high PD concentration of 25 µg mL^−1^ still showed viabilities above 95%, indicating low cytotoxicity of the nanodrug.

### 2.5 Protection Against Hepatocyte Apoptosis

ROS, the major intermediate for oxidative stress, generates excessive reactive products (e.g., 4‐HNE and MDA), which covalently attach to proteins and DNA to cause hepatocyte apoptosis and even trigger liver injury and fibrosis.[qv: 22] Therefore, the potency of nanodrug to prevent the LO2 cells from H_2_O_2_‐induced apoptosis was evaluated. First, the oxidative stress of LO2 cells was revealed by the DCFDA ROS probe. As shown in Figure S7B, Supporting Information, H_2_O_2_ stimulation markedly enhanced the ROS level of LO2 cells, whereas such effect was obviously blunted by the treatment of blank MC. Meanwhile, the LO2 cells treated with free PD also exhibited a significant decrease in ROS level. Furthermore, the LO2 cells treated with PD‐MC showed the lowest ROS level, indicating a synergistic antioxidant effect of PD and MC. Next, the apoptotic levels of LO2 cells receiving different treatments were determined. As shown in Figure S7, Supporting Information, TUNEL staining, flow cytometry of Annexin V/PI and cleaved‐caspase3 analysis obtained consistent results showing that both blank MC and PD reduced the hepatocyte apoptosis induced by H_2_O_2_. By comparison, PD‐MC appeared most potent in protecting hepatocytes against apoptosis. Additionally, the protection of PD‐MC against hepatocyte apoptosis was also confirmed in primary hepatocytes of CCl_4_‐induced mice (Figure S10A, Supporting Information). Noteworthily, the direct consumption of ROS may contribute to the anti‐apoptotic effect of the blank MC. Likely, owing to a synergistic anti‐apoptotic effect of MC and PD, PD‐MC appeared much more efficiently than PD in protecting hepatocytes against apoptosis.

### 2.6 Suppression of TLR4/NF‐κB p65 Signaling and Proinflammatory Cytokines Secretion in Macrophages

**Figure 2:**
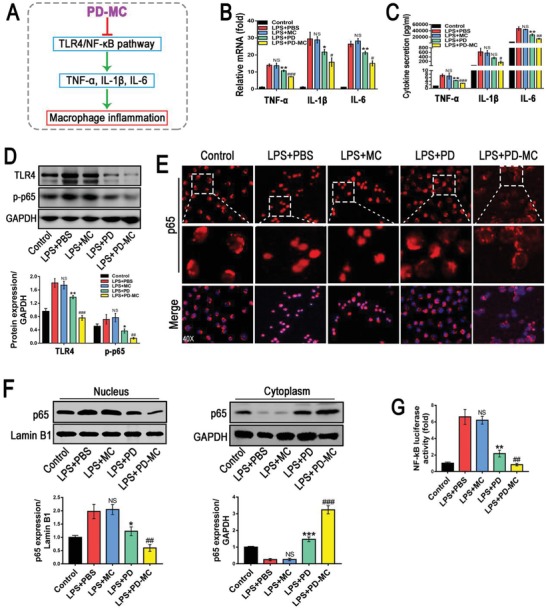
Anti‐inflammatory efficacy of PD‐MC in RAW cells challenged by LPS. A) Schematic illustration of the anti‐inflammatory mechanism of PD‐MC in macrophages. B,C) The mRNA and secretion levels of TNF‐α, IL‐1β, and IL‐6 were measured by qRT‐PCR and ELISA, respectively. *n* = 3. D) The protein levels of TLR4/NF‐κB p65 signaling pathway were measured by Western blot assay. *n* = 3. E) Nuclear translocation of NF‐κB p65 in RAW cells was revealed by immunofluorescence. F) NF‐κB p65 levels in the cytosol and nucleus were assayed by Western blot. *n* = 3. G) The NF‐κB luciferase activity in RAW cells was measured by luciferase reporter assays. *n* = 3. NS, no significance; *p < 0.05 and **p < 0.01 versus LX‐2 induced by LPS. #*p* < 0.05, ##*p* < 0.01, and ###*p* < 0.001 versus LPS induced LX2 treated with free polydatin.

The anti‐inflammatory effect of PD has been confirmed in mice with nonalcoholic steatohepatitis,[qv: 23] and hepatic macrophages are known to play a central role in initiating and perpetuating inflammation which mediated the pathogenesis of hepatic fibrosis.[qv: 24] Thus, we explored whether PD‐MC could reduce the inflammatory reaction in LPS‐activated RAW cells and primary hepatic macrophages isolated from fibrotic mice by measuring the proinflammatory cytokines (IL‐1β, IL‐6, and TNF‐α). RT‐PCR and ELISA results evidenced that even PD alone could significantly inhibit the transcription and secretion levels of proinflammatory cytokines (**Figure** **2**B,C; Figure S10B, Supporting Information), and such anti‐inflammatory effect was further strengthened by PD‐MC. Thus, the polymeric micelle allowing for ROS scavenging may provide additional anti‐inflammatory effect in liver fibrosis treatment with PD.[qv: 16] Although the undesirable inflammation induced by nanoparticles remains a common challenge nowadays,[qv: 13] the macrophages treated with blank MC showed negligible change in the inflammatory response, which could be attributed to the ROS‐consuming capacity of our micelle.[qv: 25]

TLR4, responsible for recognizing LPS deriving from Gram‐negative bacteria, collaborates with its downstream intracellular NF‐κB signals to detonate the inflammatory cytokine production in liver injury.[qv: 26–28] Consistent with the aforementioned inhibition of inflammatory cytokine production, PD‐MC also remarkably suppressed the TLR4 expression in LPS‐activated RAW cells and primary hepatic macrophages (Figure 2D; Figure S10C, Supporting Information). Moreover, according to the results of immunofluorescence staining and Western blot assays, PD‐MC behaved highly effectively in abrogating NF‐κB p65 phosphorylation and reversing the nucleus translocation of NF‐κB p65 in LPS‐activated RAW cells (Figure 2E,F). The endogenous NF‐κB transcriptional activity was also evaluated in order to gain insight into the mechanism how PD‐MC regulated inflammation. As shown in Figure 2G, after the transfection of p65‐Luc reporter plasmid, the RAW cells treated with PD‐MC showed the lowest luciferase activity under LPS stimulation, which suggested that PD‐MC reduced inflammation by deactivating NF‐κB. To further illustrate the implication of TLR4 in the anti‐inflammatory effect of PD‐MC, we performed additional overexpression study in RAW cells and demonstrated that TLR4 DNA transfection significantly reversed the down‐regulation effects of PD‐MC on the TLR4/NF‐κB p65 signaling and inflammatory reaction after LPS stimulation (Figure S11B–D, Supporting Information). Taken together, PD‐MC effectively inhibited the activation of TLR4/NF‐κB p65 signaling and secretion of proinflammatory cytokines in macrophages.

### 2.7 Alleviation of Oxidative Stress to Avoid HSC Activation

**Figure 3:**
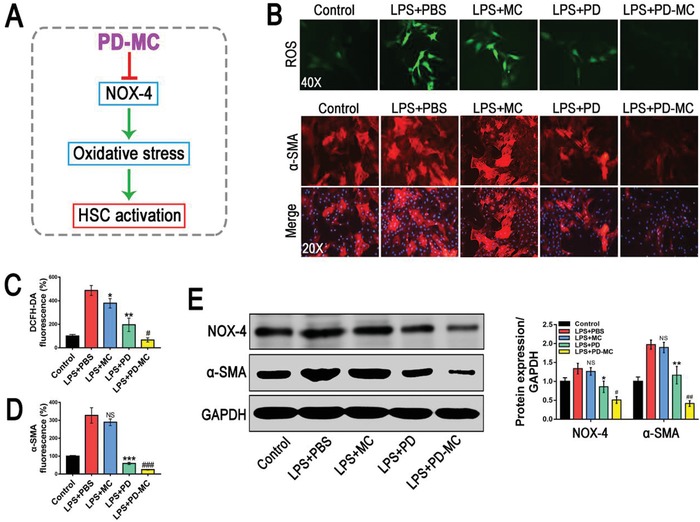
The anti‐fibrotic efficacy of PD‐MC on HSC phenotype in LX‐2 cells challenged by LPS. A) Schematic illustration of the anti‐fibrotic mechanism of PD‐MC in HSCs. B–D) The levels of oxidative stress and α‐SMA expression were measured by DCFH‐DA probe and immunofluorescence assay, respectively. *n* = 3. E) The protein expressions of NOX4 and α‐SMA were assayed by Western blot. *n* = 3. NS, no significance; *p < 0.05, **p < 0.01, and ***p < 0.001 versus LX‐2 induced by LPS. #*p* < 0.05 and ###*p* < 0.001 versus LPS induced LX2 treated with free polydatin.

Compelling evidences have revealed the close association between the increased oxidative stress and liver fibrosis.[qv: 24] During liver injuries, ROS serves as an intracellular signaling mediator of the fibrogenic action and activates the HSCs to synthesize abundant collagen. Thus, alleviation of the oxidative stress to avoid HSC activation may be a potential approach for liver fibrosis therapy. The effect of PD‐MC on oxidative stress in LPS‐activated LX‐2 cell, one well‐characterized human HSC cell line, was assessed using the DCFDA ROS probe. As revealed by the stronger fluorescence intensity, LPS stimulation markedly enhanced the ROS level of LX‐2 cells, whereas such effect was significantly weakened by the treatment of blank MC (**Figure** **3**B,C). And the LX‐2 cells treated with free PD also exhibited an obvious decrease in ROS level, which was consistent with our previous report that PD could reduce the oxidative stress during acute liver injury.[qv: 11] Meanwhile, the LX‐2 cells treated with PD‐MC showed the lowest ROS level, indicating a synergistic antioxidant action of PD and MC. Furthermore, the efficient antioxidation of PD‐MC was also demonstrated in primary HSCs isolated from CCl_4_‐induced mice (Figure S10D,E, Supporting Information). The nicotinamide adenine dinucleotide phosphate oxidase (NOX) enzyme complexes are the primary sources of endogenous ROS.[qv: 29,30] Especially, NOX4, a nonphagocytic NOX homolog which can generate ROS and activate HSCs, has been found to play a prominent role in liver fibrosis.[qv: 31] Herein, we demonstrated that PD‐MC potently down‐regulated NOX4 in LPS‐activated LX‐2 cells and primary fibrotic HSCs, which highly supported the potential NOX4 based mechanism for the ROS scavenging by PD‐MC (Figure 3E; Figure S10D,E, Supporting Information).

It was reported that the elevated levels of ROS can significantly promote liver fibrogenesis by directly activating HSCs to synthesize abundant extracellular matrix.[qv: 32] As the role of PD‐MC as ROS scavenger has been confirmed, the effect of PD‐MC on HSC activation was further investigated by analyzing the principal fibrotic marker (i.e., α‐SMA). PD‐MC functioned more effectively than free PD in deactivating LPS stimulated LX‐2 cells and primary fibrotic HSCs, and the blank MC exerted little effect (Figure 3B,D,E; Figure S10F, Supporting Information). These results implied that clearance of ROS by polymeric micelle could help improve the anti‐fibrotic effect of PD in vitro. Moreover, because NOX4 can not only generate ROS but also activate HSCs to promote liver fibrosis as mentioned above, we added an NOX4 overexpression study in the LX‐2 cells. We observed that NOX4 DNA transfection significantly reversed the inhibition of PD‐MC on the ROS level and α‐SMA expression of LX‐2 cells after LPS stimulation, which further illustrated the role of NOX4 in the deactivating effect of PD‐MC on HSCs (Figure S11F–H, Supporting Information).

### 2.8 Anti‐Inflammatory and Anti‐Fibrotic Effects

**Figure 4:**
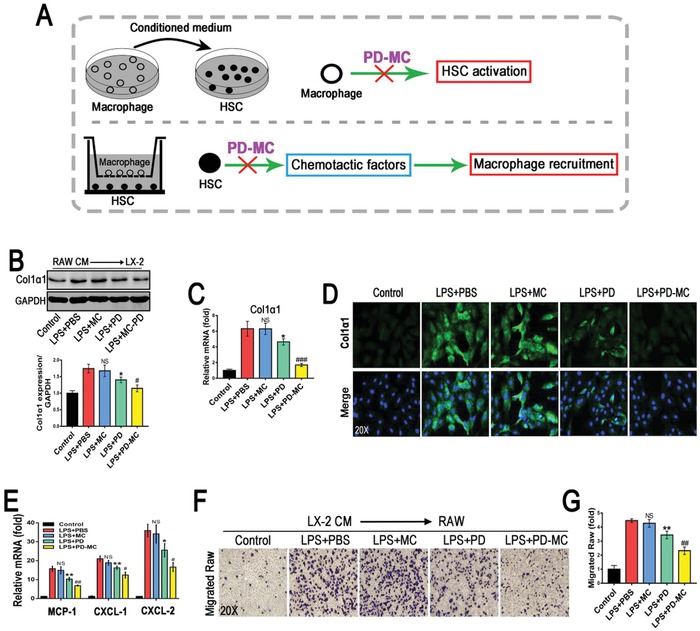
PD‐MC interrupted the interaction between activated HSCs and macrophages. A) Schematic illustration of intervening the paracrine interaction between HSCs and macrophages by PD‐MC. B–D) The effect of conditioned medium from LPS induced RAW cells with different treatments on LX‐2 cell phenotype was revealed by Western blot, qRT‐PCR and immunofluorescence assay of Collagen 1α1. *n* = 3. E) The mRNA levels of chemotactic factors, MCP‐1, CXCL‐1, and CXCL‐2, were measured by qRT‐PCR. *n* = 3. F,G) The effect of conditioned medium from LPS induced LX‐2 cells with different treatments on macrophage migration was investigated by transwell migration assays. *n* = 3. NS, no significance; *p < 0.05 and **p < 0.01 versus LX‐2 induced by LPS. #*p* < 0.05, ##*p* < 0.01, and ###*p* < 0.001 versus LPS induced LX2 treated with free polydatin.

The progression of liver fibrosis is a complex process dependent on the paracrine interaction between the proinflammatory macrophages and collagen‐synthesizing HSC, thus abolishing such interaction may result in an anti‐fibrotic effect.[qv: 33] In the present study, analyses of collagen 1α1 levels in LX‐2 cells were carried out to ascertain the effect of macrophages on the collagen synthesis of HSCs under various treatments. Consistent with the previous report,[qv: 24] the conditioned medium of RAW cells activated by LPS strongly stimulated the collagen synthesis of HSCs, whereas such effect was significantly weakened by the PD‐MC treatment (**Figure** **4**B–D). On the other hand, the activated HSCs can promote the recruitment and infiltration of macrophages through producing various chemotactic factors, which aggravates the inflammatory response during liver fibrogenesis.[qv: 34] As shown in Figure 4E, the strong inhibition of PD‐MC on the transcription of chemotactic factors (MCP‐1, CXCL‐1, and CXCL‐2) in HSCs activated by LPS was observed. Thus, the effect of the condition medium of HSCs on the macrophage migration was further checked with transwell assay. The migration of RAW cells was obviously promoted by the treatment with the conditioned medium of LX‐2 cells activated by LPS (Figure 4F,G). However, the LX‐2 cells were pretreated with PD‐MC, the macrophage migration was significantly suppressed. Interestingly, although the blank MC showed no direct effect, it did assist PD‐MC to reduce the HSC activation and macrophage migration by interrupting the paracrine interaction between two cell types, according to the better effect of PD‐MC than PD.

### 2.9 Biodistribution of PD‐MC In Vivo

**Figure 5:**
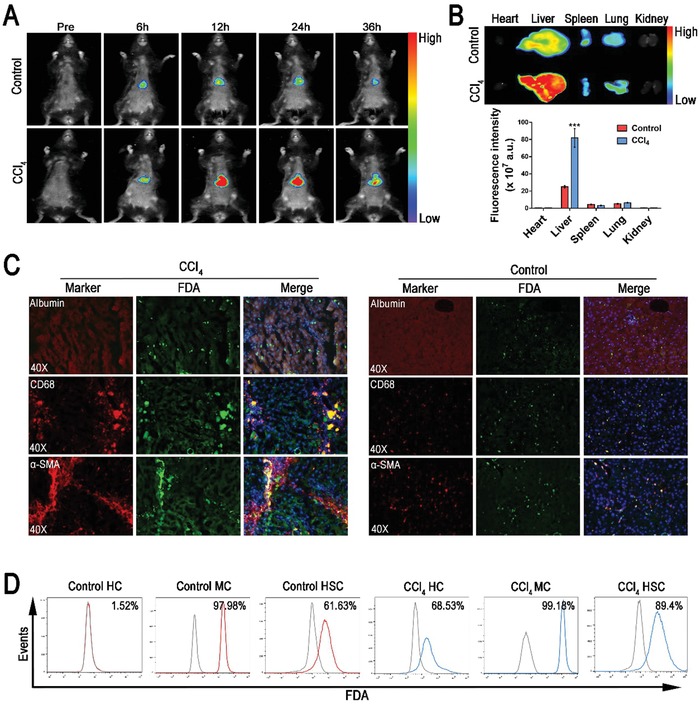
In vivo distribution of the nanomicelle. A) In vivo fluorescence imaging of mice receiving DiR‐loaded nanomicelle via i.v. injection. B) Ex vivo fluorescence imaging of the main organs from control healthy and CCl_4_ induced mice at 36 h after i.v. injection of DiR‐loaded nanomicelle. *n* = 3. C) Intrahepatic cell‐type‐specific distribution of FDA‐loaded nanomicelle in control healthy and CCl_4_ induced mice at 24 h after i.v. injection of FDA‐loaded nanomicelle. The locations of hepatocytes, macrophages and HSCs were indicated relatively by immunofluorescence staining of Albumin, CD68, and α‐SMA. D) Cellular uptake and ROS‐responsive drug release in primary hepatocytes, macrophages, and HSCs isolated from the livers of control healthy and CCl_4_ induced mice was determined by flow cytometry assay. ***p < 0.001 versus control healthy mice. HC, hepatocyte; MC, macrophage.

In vivo fluorescence imaging was performed to explore the biodistribution of the nanodrug after injection via tail vein into mice. The near‐infrared fluorescent dye 1,1′‐dioctadecyl‐3,3,3′,3′‐tetramethylindotricarbocyanine iodide (DiR) instead of PD was loaded into the micelle to enable in vivo fluorescence imaging. As shown in **Figure** **5**A, the fluorescent micelle (DiR‐MC) displayed significant hepatic accumulation in both healthy and CCl_4_ induced mice at 6 h after intravenous injection due to the entrapment by the hepatic reticuloendothelial system.[qv: 15] Nevertheless, at 12 h after injection, a higher hepatic fluorescence intensity was shown in the CCl_4_ induced mice, which was likely due to the fluorescence dequenching effect of DiR released from the micelle in response to the fibrotic microenvironment.[qv: 35,36] At 36 h after injection, a gradual decay of hepatic DiR fluorescence was observed in both healthy and CCl_4_ induced mice. The ex vivo fluorescence imaging for major organs from mice sacrificed at 36 h after injection showed consistent results (Figure 5B). The hepatic fluorescence intensities of CCl_4_ induced mice were three times higher than that of the healthy mice, once again demonstrating a much better drug release of the nanodrug under the fibrotic microenvironment. To further verify that the enhanced DiR fluorescence in liver of CCl_4_‐induced mice was caused by dye release rather than increased micelle accumulation, the biodistribution of PD in main organs at 36 h after injection of PD‐MC was detected. As shown in Figure S8, Supporting Information, the PD content showed no obvious difference in all organs of healthy and CCl_4_‐induced mice, which could be attributed to the similar pharmacokinetics of PD‐MC in the two groups of mice. All these results evidenced that the PD release from PD‐MC could be triggered by the enriched ROS in liver hepatic fibrosis.

Additionally, the cell‐specific distribution of the nanodrug in liver tissues was investigated via fluorescent imaging of Alexa Fluor 594‐stained biomarkers of different hepatic cells. Fluorescein diacetate (FDA) was used as a model hydrophobic drug instead of PD to enable fluorescent imaging under microscope. As shown in Figure 5C, the liver sections of the healthy mice showed weak FDA fluorescence mainly in non‐parenchymal cells including macrophages (CD68 positive) and HSCs (α‐SMA positive). The FDA fluorescence was undetectable in parenchymal cells, indicating the negligible uptake of nanoparticles by hepatocytes. By comparison, the liver sections of CCl_4_ induced mice displayed much stronger FDA fluorescence in both the parenchymal and non‐parenchymal cells, likely due to the drug release in response to the enriched ROS in fibrotic liver tissues. Flow cytometry was performed to quantitatively determine the fluorescence intensity of different cell types isolated from the liver (Figure 5D). In healthy mice, 99% of macrophages, 62% of HSCs, and almost no hepatocytes were fluorescence‐positive. In contrast, in CCl_4_ induced mice, 99% of macrophages, 89% of HSCs, and 69% of hepatocytes were fluorescence‐positive. These results implied that the ROS‐sensitive drug release in the fibrotic microenvironment enhanced drug uptake by both hepatocytes and HSCs.

### 2.10 Hepatoprotective Effect

**Figure 6:**
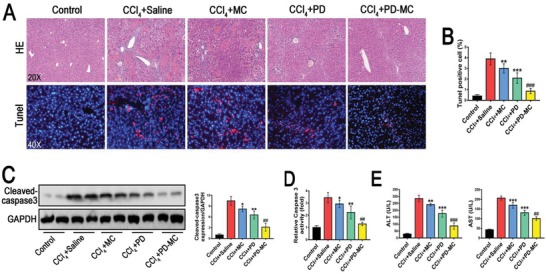
PD‐MC protected against hepatocyte apoptosis in CCl_4_ induced mice. A,B) Representative histology of H&E. Apoptotic cells in the livers were detected by means of TUNEL assay. *n* = 6. C,D) The hepatic cleaved‐caspase 3 expression (*n* = 4) and caspase 3 activity (*n* = 6) of the mice with different treatments. E) Liver function as indicated by serum levels of ALT and AST. *n* = 6. *p < 0.05, **p < 0.01, and ***p < 0.001 versus mice induced by CCl_4_. ##*p* < 0.01 and ###*p* < 0.001 versus CCl_4_ induced mice treated with free polydatin.

A high rate of hepatocyte apoptosis has been demonstrated in patients with liver fibrosis, leading to clinic application of hepatoprotective drugs for anti‐fibrotic treatment.[qv: 37] Hepatocyte apoptosis was determined to assess the hepatoprotective effect of PD‐MC in fibrotic mice. As evidenced by TUNEL assay and caspase 3 analysis, the CCl_4_ induced mice receiving PD‐MC exhibited the lowest levels of hepatocyte apoptosis (**Figure** **6**A–D). Accordingly, the least liver injury and best liver function in the PD‐MC treated mice were shown (Figure 6A,E). Interestingly, a mild relief of hepatocyte apoptosis and slight improvement of liver function were also observed in mice receiving blank MC, indicating that ROS clearance by polymer may have contributed to reduction of oxidative stress for a hepatoprotective effect.

### 2.11 Decreased Hepatic Inflammation and Oxidative Stress In Vivo

**Figure 7:**
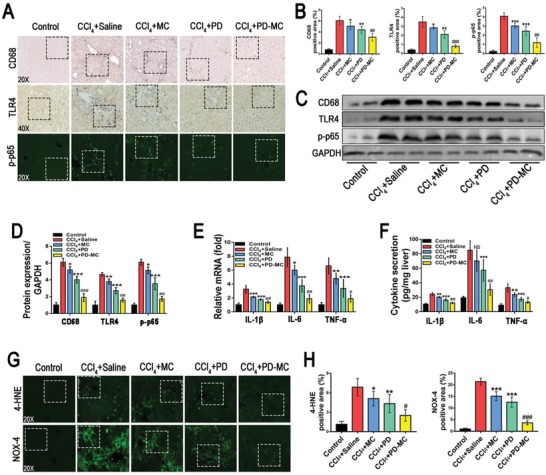
PD‐MC suppressed hepatic inflammation and oxidative stress in CCl_4_ induced mice. A–D) Hepatic expressions of CD68, TLR4, and NF‐κB p‐p65 were evaluated by immunochemical staining (*n* = 6) and Western blot assay (*n* = 4). E,F) Hepatic levels of inflammatory factors, IL‐1β, IL‐6, and TNF‐α, were measured by qRT‐PCR and ELISA. *n* = 6. G,H) Hepatic oxidative stress was evaluated by immunofluorescent staining of 4‐HNE and NOX‐4. *n* = 6. NS: no significance; *p < 0.05, **p < 0.01, and ***p < 0.001 versus mice induced by CCl_4_. #*p* < 0.05, ##*p* < 0.01, and ###*p* < 0.001 versus CCl_4_ induced mice treated with free polydatin. Dashed line squares: see Figure S15, Supporting Information, for the increased magnification.

During the initiation and progression of liver fibrosis, various injurious stimuli promote the activation and migration of the liver macrophages, which lead to the inflammatory reaction. Growing evidences are suggesting that the inflammatory reaction plays a key role in liver fibrogenesis.[qv: 8] As shown in **Figure** **7**A–F, the CCl_4_ induced mice receiving PD‐MC showed the weakest hepatic inflammation according to the fewest CD68‐positive macrophages and the least secretion of cytokines (IL‐1β, IL‐6, and TNF‐α). Noteworthily, unlike the in vitro results showing negative anti‐inflammatory effect in macrophages, the blank MC treatment reduced the hepatic inflammation in the CCl_4_ induced mice. Obviously, the anti‐inflammatory effect of blank MC is subject to the interactions between different cell types in the complex pathophysiological environment of fibrotic liver. Moreover, according to the Western blot and immunohistochemistry analyses, the CCl_4_ induced mice receiving PD‐MC showed the lowest level of hepatic TLR4/NF‐κB p65 expression, which was in line with the in vitro data of macrophages (Figure 7A–D; Figure S10C, Supporting Information). These findings provided direct evidence that the anti‐inflammatory effect of PD‐MC correlated with the inhibition of the TLR4/NF‐κB p65 signaling pathway.

Previous studies have shown that the destroyed hepatocytes generate high levels of ROS to mediate hepatic inflammation and liver fibrosis.[qv: 22] In the present study, both in vitro and in vivo experiments showed that PD‐MC not only prevented apoptosis of hepatocytes, but also exerted desirable anti‐inflammatory efficacy. It is assumable that PD‐MC may also lower the oxidative stress to cure liver injury induced by CCl_4_. In addition to the clear antioxidant effect on HSCs in vitro (Figure 3B,C; Figure S10D,E, Supporting Information), the PD‐MC treatment effectively alleviated hepatic oxidative stress in CCl_4_ induced mice as well. As shown in Figure 7G,H, compared to the CCl_4_ induced mice receiving blank MC or free PD, the mice receiving PD‐MC displayed a much lower hepatic oxidative stress as evidenced by the reduction of 4‐HNE. Moreover, the immunofluorescence assay validated that the mice receiving PD‐MC showed the lowest hepatic protein levels of NOX4, a major ROS producer mediating oxidative stress and HSC activation during liver fibrogenesis (Figure 7G,H).[qv: 32] Therefore, our results also revealed a potential NOX4 based mechanism for the antioxidant activity of PD‐MC in the fibrotic liver induced by CCl_4_.

### 2.12 Suppression of HSC Activation and Liver Fibrosis

**Figure 8:**
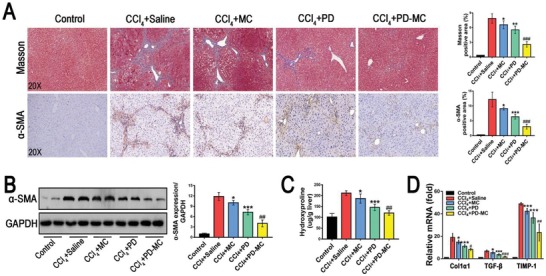
PD‐MC improved liver fibrosis in CCl_4_ induced mice. A) Representative hepatic histology of Masson and immunohistochemical staining of α‐SMA. *n* = 6. B) Hepatic α‐SMA expression was measured by Western blot assay. *n* = 4. C) Quantification of hepatic collagen accumulation was evaluated by hydroxyproline content assay. *n* = 6. D) Hepatic mRNA levels of Col1α1, TGF‐β, and TIMP‐1 were measured by qRT‐PCR. *n* = 6. *p < 0.05, **p < 0.01, and ***p < 0.001 versus mice induced by CCl_4_. #*p* < 0.05, ##*p* < 0.01, and ###*p* < 0.001 versus CCl_4_ induced mice treated with free polydatin.

As a result of ongoing hepatocyte death, inflammatory reaction and oxidative stress, the quiescent HSCs transdifferentiate into an activated phenotype, which synthesize abundant extracellular matrix and play a central role in liver fibrogenesis. Antifibrotic strategies targeting the activated HSCs have been investigated in previous study.[qv: 38] On the basis that PD‐MC impeded the HSC activation in vitro (Figure 3B,D; Figure S10F, Supporting Information), antifibrotic effect of nanodrug in the CCl_4_ induced mice was further explored. As shown in **Figure** **8**A,B, according to the α‐SMA assessment and Masson staining, the CCl_4_ induced mice treated with PD‐MC showed significantly reduced levels of both HSC activation and hepatic collagen accumulation. Furthermore, the reduction of liver fibrosis was confirmed in mice receiving PD‐MC by quantitatively analyzing hydroxyproline and fibrotic markers such as, Col1α1, TGF‐β, and TIMP‐1 (Figure 8C,D). Previous reports have highlighted the benefits of ROS‐reacting nanoparticles in liver fibrosis treatments.[qv: 16] In the present study, we also observed obviously reduced HSC activation and hepatic collagen accumulation in the mice treated with blank MC, underlining the potential of ROS‐reacting nanoparticles to serve as therapeutic agents for liver fibrosis. Thus, PD‐MC achieved better anti‐fibrotic efficacy than free PD in CCl_4_ induced mice (Figure 8A,B), a finding that was reasonable because PD‐MC not only realized the liver‐targeted delivery of PD acting on multiple injured liver cells (Figure 5C,D), but also provided anti‐fibrotic benefits by removing ROS.

Noteworthily, in our current study, we also observed that two weeks of PD‐MC treatment showed an anti‐fibrotic effect in mice which have already developed chronic liver fibrosis after 6 weeks of CCl_4_‐induction (Figure S13, Supporting Information). Hence, further study will be performed in future for a better understanding of the therapeutic potential and mechanism of PD‐MC in advanced liver fibrosis (e.g., cirrhosis or portal hypertension).

### 2.13 Side Effects In Vivo

As shown in Figure S9A, Supporting Information, H&E staining showed that PD‐MC treatment caused no structural damage to major organs in CCl_4_ induced mice, indicating low side effects in vivo. It is worth noting that, according to the results of H&E staining and analyses of renal serum function markers (creatinine and urea), CCl_4_ induced mice receiving PD showed mild improvement of renal function (Figure S9, Supporting Information), and such a nephroprotective effect of PD was consistent with a previous report.[qv: 39] However, PD‐MC treatment was unable to alter the renal function of CCl_4_ induced mice, likely because of the extremely low accumulation of PD‐MC in kidney (Figure 5B).

## 3 Discussion

Liver fibrosis, a major liver disease with different etiologies, is seriously threatening human health but still lacking reliable therapeutic options.[qv: 40,41] In the current study, the ROS and pH dual‐sensitive polydatin‐loaded micelle (PD‐MC) was developed as a potent therapeutic agent for liver fibrosis. PD is a promising herbal medicine with various beneficial effects including antioxidant defense, anti‐inflammatory effect and immunomodulatory activity. In recent years, PD has attracted tremendous attention due to its remarkable therapeutic effects in various diseases, such as respiratory infection, parkinson disease, and cancers.[qv: 42–45] However, its applications in treatments of liver diseases were hampered by the extremely low solubility, low hepatic drug bioavailability and non‐specific drug uptake in other organs.[qv: 12,39] Nowadays, microenvironment‐sensitive nanocarriers have demonstrated great potentials in increasing the bioavailability of hydrophobic agents, thereby improving the therapeutic outcomes in tumors, atherosclerosis, acute liver failure and periodontitis.[qv: 46–49] Herein, we developed a polymer‐based PD delivery system which enabled the intravenous administration and specifically released drug in the microenvironment of fibrotic liver to target different types of hepatic cells. The work aimed at offering a proof of concept to design PD‐MC with ROS and pH dual‐sensitivity to realize site‐specific drug release in the ROS‐rich environment of fibrotic liver and the acidic lysosomal compartments. The PD‐MC specifically released drug in pathological microenvironment of fibrotic liver to target various types of liver cells, resulting in a highly effective therapy and minimal side effects in liver fibrosis treatment.

During the initiation of fibrogenesis, hepatocyte death resulting from chronic liver injury promotes the release of apoptotic bodies which recruit and activate hepatic macrophages.[qv: 50–52] Consequently, the activated hepatic macrophages produce a large amount of inflammatory cytokines and ROS, which not only aggravate the hepatocyte death by a metabolic flux disruption, but also activate the quiescent HSCs into myofibroblast‐like phenotype.[qv: 53–55] Our results demonstrated the hepatoprotective capacity of PD‐MC in H_2_O_2_ stimulated hepatocytes and CCl_4_ induced mice. Interestingly, the blank MC also exerted anti‐apoptotic effect on hepatocytes, which could be attributed to the direct consumption of ROS by the PPBEM block of polymer.[qv: 16,49] Aside from the hepatoprotective effect, the PD‐MC also displayed potent anti‐inflammatory activity in macrophages and fibrotic liver, which was accompanied by a down‐regulation of TLR4 expression. It is well known that TLR4 up‐regulates both proinflammatory and profibrogenic cytokines through activating its downstream signaling, nuclear factor κB (NF‐κB).[qv: 26–28] Herein, we also provided clear evidences that PD‐MC suppressed the NF‐κB p65 signaling of macrophages and the livers of CCl_4_‐induced mice. These results implied that the anti‐inflammatory effect of PD‐MC was likely related to shutting down the TLR4/NF‐κB p65 signaling pathway during liver fibrogenesis. It was worth noting that the blank MC mildly reduced the hepatic inflammation in CCl_4_ induced mice, indicating that the MC may not only consume ROS to reduce oxidative stress, but also exerted anti‐inflammatory effect as supported in other researches.[qv: 16,56,57] Nevertheless, as evidenced by the in vitro data, the blank MC showed negative anti‐inflammatory effect in the LPS induced RAW cells and the primary macrophages isolated from the CCl_4_ induced mice. The different in vitro and in vivo results suggested that the ROS‐consuming MC might have exerted anti‐inflammatory effect also modulated by the interactions between different cell types in the pathophysiological environment of fibrotic liver. Thus, the blank MC synergistically improved the anti‐inflammatory activity of the delivered PD, leading to a remarkable down‐regulation of hepatic inflammation in the fibrotic mice receiving PD‐MC.

Upon continuous liver injury, the ongoing hepatocyte death and inflammatory reaction activate the HSCs to transdifferentiate into myofibroblasts which play a key role in liver fibrosis by producing ECM.[qv: 58,59] Various mediators, including ROS, cytokines, matrix stiffness and growth factors, drive the activation of HSCs in an autocrine or paracrine fashion. In particular, previous studies have highlighted the significance of NOX4‐mediated ROS overproduction to deteriorate liver fibrosis by activating HSCs.[qv: 29,30] Our study clearly demonstrated that PD‐MC suppressed NOX4 expression and ROS production in HSCs, as well as in the livers of CCl_4_ induced mice. Then, we further found that PD‐MC dramatically inhibited the activation and collagen synthesis of HSCs. Additionally, the blank MC also halted the progression of liver fibrosis to some extent, which can be explained by its antioxidant and anti‐inflammatory activities as mentioned above. The above results highlighted that, for liver fibrosis treatment, PD‐MC is advantageous over free PD in two aspects, which includes the liver‐targeted drug delivery and the synergistic antifibrotic effect of the nanocarrier.

The progression of liver fibrosis is a complex process involving the interaction among various types of liver cells. During liver fibrogenesis, hepatic macrophages promote the activation and survival of HSCs by releasing multiple profibrogenic mediators. In return, activated HSCs provide chemotactic signals that further irritate the recruitment and migration of macrophages to exacerbate the inflammatory reaction.[qv: 60,61] Such paracrine interaction between HSCs and hepatic macrophages behaves as an important executor in liver fibrogenesis. In co‐cultural experiments, after PD‐MC treatment, the LX‐2 cells showed a significantly weakened stimulation on macrophage migration, likely due to the reduced secretion of chemotactic factors. On the other hand, the RAW cells treated with PD‐MC displayed an obviously declined stimulation on HSC activation. Thus, the blockage of the paracrine interaction between HSCs and macrophages was identified as another important mechanism mediating the antifibrotic effect of PD‐MC.

Liver sinusoidal endothelial cells (LSECs) are the most abundant non‐parenchymal hepatic cells which constitute the sinusoidal capillary channels of the liver.[qv: 62] It has been demonstrated that the reduced nitric oxide (NO) availability and increased thromboxane A_2_ (TXA_2_) production are the main factors mediating the dysfunction of LSECs, which promotes the development of liver fibrosis and cirrhotic portal hypertension.[qv: 63,64] However, our data revealed that PD‐MC had no obvious influence on the NO availability and TXA_2_ production of LSECs, which may indicate that the anti‐fibrotic effect of PD‐MC was independent of modulating the LSEC function (Figure S12, Supporting Information).

## 4 Conclusion

A polydatin‐encapsulated micelle (PD‐MC) with ROS and pH dual‐sensitivity is developed based on block polymer PEG‐b‐P(PBEM‐*co*‐DPA) for the liver fibrosis therapy. PD‐MC achieves highly efficient delivery of polydatin to liver, and specifically releases drug in response to the fibrotic microenvironment to target multiple types of hepatic cells. Both in vitro and in vivo results demonstrate that PD‐MC can effectively ameliorate liver fibrosis by suppressing inflammatory reaction and oxidative stress, preventing hepatocyte apoptosis and averting activation of macrophages and HSCs. Our study shows the great potential of PD‐MC as a potent therapeutic agent for liver fibrosis treatment.

## 5 Experimental Section

##### Preparation of the Dual Stimuli‐Responsive Micelle

PEG‐P(PBEM‐*co*‐DPA) (20 mg) and hydrophobic agent polydatin were dissolved in 2 mL DMSO/CHCl_3_ (v/v, 1:4) mixture solvent. Afterward, the mixture was added dropwise into 10 mL purified water under sonication (VCX130, Sonics, USA, 20 kHz, 40% power level). The CHCl_3_ was removed by vacuum using a rotary evaporator, then the micelle solution was dialyzed (MWCO: 14 kDa) against purified water to remove free PD and DMSO solvent. Finally, the micelle solution was filtered through the 0.45 µm filter membrane to remove the large aggregate, and the PD‐loaded micelle (NP‐PD) was obtained. The characterization of the dual stimuli‐responsive micelle was described in the Supplementary Information.

##### Cell Isolation and Culture

The murine macrophage cell line RAW264.7 (RAW) was provided generously by Teng Wu (Department of Pathophysiology, Nanjing Medical University, Nanjing 210000, China). The well‐characterized cell line derived from human HSC, LX‐2 and human hepatocyte cell line LO2 were generously provided as a gift by Professor Qi Zhang (the Third Affiliated Hospital of Sun Yat‐sen University, Guangzhou 510630, China). Primary hepatocytes, HSCs and macrophages were isolated from the mouse livers and identified as described in our previous studies.[qv: 34,65] The primary cells, RAW, LO2, and LX‐2 cells were cultured in Dulbecco's modifier Eagle's medium (DMEM) supplemented with 10% fetal bovine serum (FBS) and 1% penicillin‐ streptomycin in a humidified atmosphere containing 5% CO2 at 37 °C.

##### Cell Treatment

Before the drug intervention, cells were synchronized in serum‐free DMEM for 24 h. For LO2 cells, the medium was then replaced by 1) serum‐free DMEM, 2) serum‐free DMEM with 100 × 10^‐6^ m H_2_O_2_, 3) serum‐free DMEM with 100 × 10^‐6^ m H_2_O_2_ and 10 × 10^‐6^ m polydatin, 4) serum‐free DMEM with 100 × 10^‐6^ m H_2_O_2_ and polydatin‐loaded nano‐micelle (PD‐MC, loaded with 10 × 10^‐6^ m polydatin), 5) serum‐free DMEM with 100 × 10^‐6^ m H_2_O_2_ and blank nano‐micelle (MC, without polydatin). For RAW and LX‐2 cells, the medium was then replaced by 1) serum‐free DMEM, 2) serum‐free DMEM with 100 ng mL^−1^ LPS, 3) serum‐free DMEM with 100 ng mL^−1^ LPS and 10 × 10^‐6^ m polydatin, 4) serum‐free DMEM with 100 ng mL^−1^ LPS and polydatin‐loaded nano‐micelle (PD‐MC, loaded with 10 × 10^‐6^ m polydatin), 5) serum‐free DMEM with 100 ng mL^−1^ LPS and blank nano‐micelle (MC, without polydatin).

##### Macrophage Migration Assay

The paracrine effect of HSCs (LX‐2 cells) on the migration of macrophages (RAW cells) was investigated by the Boyden chamber transwell assay. Briefly, the LX‐2 cells were first seeded in the lower chamber plate for 12 h to allow attachment. Then LX‐2 cells were treated as described in the “Cell Treatment” section and incubated for 24 h. After the replacement of the culture media by regular culture media for another 24 h incubation, RAW cells in regular culture media were seeded on the upper chamber to start the migration assay. 24 h later, migrated RAW cells were fixed with 4% paraformaldehyde and stained by crystal violet staining solution. The images were captured using Olympus IX71 microscope (Olympus Co., Tokyo, Japan) and then the chambers were washed by acetic acid solution. Finally, the eluent was measured by a microplate reader at 570 nm for the quantitative determination of the numbers of migrated cells.

##### Luciferase Reporter Assay

RAW cells were transfected with plasmid NF‐κB using Lipofectamine 3000 (Invitrogen, USA). 12 h after transfection, the cells were culture in DMEM containing 10% FBS overnight and then treated as described in the “Cell Treatment” section and incubated for 24 h. The cells were lysed and luciferase activity was determined by a dual luciferase assay system (Promega Corp., USA). Renillaluciferase luciferase activity was used for normalization.

##### Induction of Hepatic Fibrosis

Male C57BL/6 mice were obtained from the Center of Experimental Animal of Sun Yat‐sen University. All animal procedures were conducted in accordance with the National Institutes of Health Guide for the Care and Use of Laboratory Animals and approved by the Ethics Committee on the Care and Use of Laboratory Animals of Guangdong Pharmaceutical University (Guangzhou, China). Liver fibrosis was induced in C57BL/6 mice by intraperitoneal injections of CCl_4_ (1 mL per kg body weight, dissolved in corn oil at a ratio of 1:4, twice a week) for 6 weeks. Mice in untreated groups were administrated with the same volume of corn oil as healthy control.

##### Treatment of Liver Fibrosis

For therapeutic study, after 3 weeks of CCl_4_ administration, the C57BL/6 mice were treated with the following samples every other day for another three weeks:1) 40 mg per kg blank nano‐micelle (MC) through tail vein injection; 2) 2.5 mg per kg polydatin (PD) suspended in saline through intraperitoneal injection; 3) 40 mg per kg polydatin loaded nano‐micelle (PD‐MC) through tail vein injection (containing 2.5 mg per kg polydatin). The same volume of saline was administered to the mice through tail vein injection in both the healthy control group and the fibrotic model group. In our preliminary experiment (data not shown), the results of Sirius red staining and hydroxyproline assay indicated that the therapeutic effects of polydatin on liver fibrosis were not significantly boosted by higher doses of polydatin. That is, the groups treated with different doses of polydatin (2.5 and 5 mg per kg) showed similar reductions in liver fibrosis. Thus, in the present study, we chose 2.5 mg per kg polydatin to treat mice.

##### Statistical Analysis

The results were expressed as mean ± standard deviation (SD). Statistical differences between two groups were analyzed by the unpaired student's t test and differences between multiple groups were analyzed by one‐way ANOVA with Bonferroni correction (GraphPad Prism 5.0, San Diego, CA, USA). *p* < 0.05 was considered statistically significant.

## Conflict of Interest

The authors declare no conflict of interest.

## Supporting information

Supporting Information
