# Supplementary material for: Nanodrug with ROS and pH Dual‐Sensitivity Ameliorates Liver Fibrosis via Multicellular Regulation
Source: Adv Sci (Weinh). 2020 Feb 14;7(7):1903138. doi: 10.1002/advs.201903138 (PMC7140994; doi:10.1002/advs.201903138)
Supplement: Supplementary file 1 — Supporting Information [file ADVS-7-1903138-s001.pdf]

Copyright WILEY-VCH Verlag GmbH & Co. KGaA, 69469 Weinheim, Germany, 2020.

ADVANCED SCIENCE
Open Access

Supporting Information

for *Adv. Sci.,* DOI: 10.1002/advs.201903138

Nanodrug with ROS and pH Dual-Sensitivity Ameliorates Liver Fibrosis via Multicellular Regulation

Liteng Lin, Hengye Gong, Rui Li, Jingjun Huang, Mingyue Cai, Tian Lan, Wensou Huang, Yongjian Guo, Zhimei Zhou, Yongcheng An, Zhiwei Chen, Licong Liang, Yong Wang, Xintao Shuai,* and Kangshun Zhu**

Nanodrug with ROS and pH dual-sensitivity ameliorates liver fibrosis via multicellular regulation

Liteng Lin, Hengye Gong, Rui Li, Jingjun Huang, Mingyue Cai, Tian Lan, Wensou Huang, Yongjian Guo, Zhimei Zhou, Yongcheng An, Zhiwei Chen, Licong Liang, Yong Wang,* Xintao Shuai,* Kangshun Zhu *

Dr. L. Lin, R. Li, Dr. J. Huang, Dr. M. Cai, W. Huang, Y. Guo, Z. Zhou, Y. An, Z. Chen, L. Liang, Prof. K. Zhu

Laboratory of Interventional Radiology, Department of Minimally Invasive Interventional Radiology, and Department of Radiology, The Second Affliated Hospital of Guangzhou Medical University, Guangzhou 510260, China

*E-mail: zhksh010@163.com

H. Gong, Prof. X. Shuai

PCFM Lab of Ministry of Education, School of Material Science and Engineering, Sun Yat-Sen University, Guangzhou 510275, China

*E-mail: shuaixt@mail.sysu.edu.cn

Dr. T. Lan

School of Pharmacy, Guangdong Pharmaceutical University, Guangzhou 510006, China

Dr. Y. Wang

College of Chemistry and Materials Science, Jinan University, Guangzhou 510632, China

*E-mail: wangy488@mail.sysu.edu.cn

Materials: S-1-dodecyl-S′-(α,α′-dimethyl-α″-acetic acid) trithiocarbonate (DDAT) was synthesized as reported before [1]. 4-(Hydroxymethyl)phenylboronic acid, pinacol, N,N'-Carbonyldiimidazole, 2-hydroxyethyl methacrylate, 4-dimethylaminopyridine, azodiisobutyronitrile, dicyclohexylcarbodiimide, N-hydroxysuccinimide (NHS), dimethyl sulfoxide (DMSO) and dimethylformamide (DMF) and 2-(diisopropyl amino)ethyl methacrylate (DPA) were purchased from Sigma-Aldrich (St. Louis, CA) and purified using alkaline aluminum oxide column chromatography before use. Dialysis bag (MWCO: 3.5 kDa and 14 kDa) was purchased from Shanghai Green Bird Technology Development Co., Ltd. Tetrahydrofuran (THF), Chloroform ($CHCl_3$), petroleum ether and acetic ether were dried over CaH_2 and then distilled under ambient pressure. Dioxane, diethyl ether was of analytical grade and purchased from Guangzhou Chemical Reagent Factory, China. Polydatin (PD), lipopolysaccharide (LPS), hydrogen peroxide (H_2O_2) and dichlorofluorescin diacetate (DCFH-DA) were purchased from Sigma-Aldrich (St. Louis, CA). Carbon tetrachloride (CCl_4) and corn oil were purchased from Aladdin (Shanghai, China). Dulbecco’s modified Eagle’s medium (DMEM), fetal bovine serum (FBS), penicillin/streptomycin solution, Trypsin-EDTA, phosphate-buffered saline (PBS), TRIzol reagent, dihydroethidium (DHE), Alexa Fluor 488 and 594-conjugated secondary antibodies and anti-CD31 antibody were from Invitrogen Life Technologies (Carlsbad, CA, USA). PrimeScript RT-PCR Kit was purchased from Takara (Dalian, China). RIPM lysis buffer and One Step TUNEL Apoptosis Assay Kit were purchased from Beyotime Biotechnology (Shanghai, China). The Cell Counting Kit-8 (CCK-8) was purchased from Dojindo

Laboratories (Kumamoto, Japan). Anti-α-SMA, anti-CD68, and anti-TLR4 antibodies were from Boster Biological Technology Co, Ltd. (Wuhan, China). Anti-4HNE, anti-NOX4 and anti-Desmin antibodies were from Abcam Inc. (Cambridge, UK). Anti-Albumin antibody was from Proteintech Group Inc. (Proteintech, Rosemont, USA). Anti-Cleaved-caspase3, anti-NF-κB p-p65 and anti-NF-κB-p65 antibodies were from Cell Signaling Technology Inc. (Beverly, MA, USA). Anti-GAPDH antibody, horseradish peroxidase (HRP) conjugated AffiniPure goat anti-mouse IgG and anti-rabbit IgG were purchased from Zhongshan Golden Bridge Biotechnology Co. Ltd. (Beijing, China). Enhanced chemiluminescence (ECL) substrate for HRP detection were obtained from Pierce Thermo Scientific (Rockford, USA). Enzyme linked immunosorbent assay (ELISA) kits for IL-1β, IL-6 and TNF-α were purchased from Cusabio Biotech Co. (Wuhan, China). Dual-Luciferase Reporter Assay Kit was purchased from Promega Biotech Co. (Promega, USA). Annexin V-FITC kit and caspase-3 activity kit were from BestBio (Shanghai, China). Alanine aminotransferase (ALT), aspartate aminotransferase (AST), creatinine (Cr), blood urea, and hydroxyproline assay kits were purchased from Nanjing Jiancheng Bioengineering Institute (Nanjing, China).

Synthesis of CDI-activated pinacol boronic ester: 4-(Hydroxymethyl) phenylboronic acid (6 g, 39.4 mmol), pinacol (5.6 g, 47.7 mmol) and sodium sulfate (6 g) were charged into 100 mL flask bottle, then dissolved in THF (50 mL) solvent. The reaction were kept stirring at room temperature for 12 h under nitrogen protection. Then the mixture was filtered, concentrated, re-dissolved in ethyl acetate. The

solution was precipitated into cold excessive diethyl ether and washed with diethyl ether twice. Finally, the precipitated was vacuum-dried to obtain the product 4-(hydroxymethyl)phenylboronic acid pinacol ester (6.3 g, yield: 68.4%). Subsequently, 4-(hydroxymethyl)phenylboronic acid pinacol ester (5 g, 21.3 mmol), CDI (3 g, 48.6 mmol) were charged into 100 mL flask bottle, then dissolved in anhydrous $CHCl_2$ (30 mL) solvent. After stirring for 6 h at room temperature, the solution was concentrated, re-dissolved in ethyl acetate, and washed with purified water three times. The organic phase was dried over anhydrous sodium sulfate, filtered, then vacuum-dried to obtain CDI-activated pinacol boronic ester (4.2 g, 60.8 %).

Synthesis of ROS-responsive monomer 2-((((4-(4,4,5,5-tetramethyl-1,3,2-dioxaborolan-2-yl)benzyl)oxy)carbonyl)oxy)ethyl methacrylate (PBEM): CDI-activated pinacol boronic ester (4 g, 12.2 mmol), 2-hydroxyethyl methacrylate (1.9 g, 15 mmol) and DMAP (18 mg, 0.15 mmol) were dissolved in anhydrous $CHCl_3$ (30 mL) and stirred for 12 h at room temperature. The mixture was diluted in ethyl acetate (60 mL), then washed with purified water three times (10 mL each time). The organic phase was dried over anhydrous sodium sulfate, filtered, and concentrated to obtain the H_2O_2-responsive monomer PBEM.

Synthesis of polymer PEG-P(PBEM-co-DPA): The copolymer PEG-P(PBEM-co-PEM)) was prepared using reversible addition-fragmentation chain transfer (RAFT) copolymerization of PBEM and DPA monomers with 2-(dodecylthiocarbonothioylthio)-2-methylpropionic acid (DDAT) as the chain

transfer agent. Firstly, DDAT (0.1 g, 0.27 mmol), DPA monomer (1.61 g, 7.56 mmol), PBEM monomer (1.57 g, 4.05 mmol) and AIBN (5.7 mg 0.035 mmol) were dissolved in 10 mL 1,4-dioxane, then charged into 50 mL Schlenk flask. The mixture was blown with nitrogen for 30 min to remove oxygen, then the bottle was sealed and kept stirring at 80 °C for 8 h. After cooling in ice quickly, the solution was dialyzed (3.5 kDa) against 1,4-dioxane for 3 days. Then the solution was evaporated to obtain the product P(PBEM-co-DPA)-DDAT (2.44 g, 74.3 %). The collected product and excessive AIBN were dissolved in 10 mL 1,4-dioxane and blown with nitrogen for 30 min, then sealed and heated to 80 °C for 8 h. After cooling in ice quickly, the solution was dialyzed (3.5 kDa) against 1,4-dioxane for 3 days. The light yellow product P(PBEM-co-DPA) was finally obtained after evaporation (1.8 g, yield: 76.9 %). P(PBEM-co-DPA) (1.0 g, 83 μmol), NHS (34.5 mg, 150 μmol) and EDC•HCl (57.3 mg, 150 μmol) were dissolved with 10 mL anhydrous DMSO in a 50 mL Schlenk flask, the reaction was kept stirring at room temperature for 4 h. Afterwards PEG-NH_2 (0.2 g, 100 μmol) was added into the flask, and kept stirring at room temperature for 24 h. After reaction, the mixture solution was dialyzed (3.5 kDa) against 1,4-dioxane for 3 days and methanol for 2 days. The solvent was evaporated to obtain the yellow product PEG-P(PBEM-co-DPA) (1.1 g, yield: 91.7 %).

Characterization of polymer PEG-P(PBEM-co-DPA): ^{1}H-NMR spectra of the PBEM monomer and polymers were obtained using an AVANCE III 400 MHz spectrometer with DMSO-d_6 as the solvent. The molecular weight distributions of the polymers were tested by gel permeation chromatography (GPC) system (Waters

Breeze, USA) containing an UltrahydrogelTM 500 column and an Ultrahydrogel^{TM} 250 column. The GPC measurements were carried out with PEG as a pre-established calibration standard, and DMF as an eluent at a flow rate of 1.0 mL min-1, and with polystyrene as a pre-established calibration standard. Besides, PEG-P(PBEM-co-DPA) was dissolved in H_2O_2 solution (5 mM), the solution was freeze-dried at certain time points (1 h, 6 h, 10 h, 24 h), the powder obtained after freeze-dry was redissolved in D_2O, and the ^{1}H NMR spectra were recorded.

Characterization of the dual stimuli-responsive micelle: After the micelle solution was obtained, the morphology of the micelle at four different condition (pH 7.4, pH 5.0, pH 7.4 + 0.1 mM H_2O_2, pH 5.0 + 0.1 mM H_2O_2) was analyzed using the transmission electron microscopy (Hitachi JEM-1400, Japan) operated at 120 kV. Briefly, the micelle solution was diluted to 0.5 mg·mL^{-1} with different conditions. After 3 h, the micelle solution (10 µL) obtained was dropped on a copper grid coated with amorphous carbon. The copper gried was dried, then stained with uranyl acetate solution (10 µL). After blotting off the remaining solution with filter paper, the gird was dried overnight to obtain the TEM samples. The sizes and zeta potentials of micelles were determined using the dynamic light scattering (DLS) measurement (Brookhaven 90 Plus/BI-MAS, USA) with a detection angle of scattered light at 90° and 15°, respectively.

Serum stability assay: To evaluate the serum stability of nanodrug used in the biological tests, micelle solution of PD-MC was incubated at 37 °C in PBS supplemented with 10% fetal bovine serum (FBS). The changes of size and zeta

potential of nanodrug was monitored up to 24 h using DLS at 25 °C.

Detection of H_2O_2 consumption of polymer: Catalase-like activity of PEG-P(PBEM-co-DPA) was determined by the Góth method [2]. Briefly, 20 mL of hydrogen peroxide (0.055, 0.11 or 1.1 mM) was incubated with 2 mL of polymer solution (molar concentration of PBEM unit: 1.1 mM) at 37 °C. The final regents were fixed at H_2O_2, 0.05, 0.1 or 1 mM; PBEM unit of polymer, 1 mM; and ammonium molybdate, 450 µM at predetermined time points, 1 mL mixed solution was collected and terminated by adding 1 mL of ammonium molybdate (1 mM). Also, the control group was obtained by replacing the polymer solution with purified water. The residual H_2O_2 would react with ammonium molybdate to form a stable yellow complex. Thus, the H_2O_2 concentration can be determined by the absorbance of the complex at 375 nm using a pre-established standard curve. The H_2O_2 consumption was calculated based on the difference of residual H_2O_2 between the experiment group with polymer and the control group without polymer at various hydrogen peroxide concentrations.

Loading content of polydatin: The loading content of polydatin in the drug-loaded micelle was determined using the UV-Vis spectrophotometry. Briefly, the micelle solution (0.5 mL) was freeze-dried and weighted, then redissolved in 1 mL DMSO/$CHCl_3$ (v/v, 1:1). PerkinElmer UV750 spectrophotometer was used to measure the absorbance intensity of polydatin at 358 nm. After that, the Loading content of polydatin was calculated according to pre-established calibration curve.

Fluorescence intensities change: To analyze the dual stimuli-responsive behavior

of micelle, the fluorescence spectra of micelle solution under four different conditions (pH 7.4, pH 5.0, pH 7.4 + 0.1 mM H_2O_2 and pH 5.0 + 0.1 mM H_2O_2) were determined. Briefly, 1 mL micelle solution was diluted in 4 mL PBS solution under different conditions (pH 7.4, pH 5.0, pH 7.4 + 0.1 mM H_2O_2 and pH 5.0 + 0.1 mM H_2O_2). The diluted solution was kept static for 3 h after shaking. The fluorescence spectra were recorded using Shimadzu RF-5301 Fluorophotometer. Excited wavelength: 458 nm. Emission wavelength range 350-510 nm.

In vitro drug release assay: To study the in vitro drug release behavior of the dual stimuli-responsive micelle, the drug release tests were performed under the same conditions above (pH 7.4, pH 5.0, pH 7.4 + 0.1 mM H_2O_2 and pH 5.0 + 0.1 mM H_2O_2). Briefly, 1 mL micelle solution was diluted in 4 mL PBS solution under corresponding conditions. Then the diluted solution was dialyzed (MWCO: 14 kDa) against 20 mL same PBS at 37 °C in an incubator shaker. At specific time points, 5 mL solution outside was collected to measure the concentration of polydatin afterwards, then replenished with 5 mL fresh PBS with the same pH. The drug release studies were measured in triplicate for each pH values.

Cytotoxicity assay: The RAW, LO2 and LX-2 cells were respectively seeded into 96-well plates at a density of 5 × 10^3 cells per well and incubated for 12 h. Then, the medium was added with the following samples: Ploydatin and Polydatin-loaded nanomicelle (PD-MC) at various Ploydatin concentrations (3.125, 6.25, 12.5, 25, 50, 100 μg/mL). After 48 h of culture, each well was added with 10 μL of CCK-8 solution, then the cells were incubated for another 3 h. Finally, the absorbance at 450 nm was tested by a microplate reader (Tecan, Crailsheim, Germany).

In vivo and ex vivo fluorescence imaging: For *in vivo* fluorescence imaging, the healthy control and the fibrotic control C57BL/6 mice were injected with DiR-loaded nano-micelle through tail vein at a DiR dose of 1 mg/kg body weight. The fluorescence images were captured on *in vivo* imaging system (Carestream, NY, USA) at determined post-injection time. For *ex vivo* fluorescence imaging, the healthy control and the fibrotic control C57BL/6 mice were injected with FDA-loaded nano-micelle through tail vein at a FDA dose of 1 mg/kg body weight. 24 h after injection, the mice were sacrificed and perfused with PBS in the blood circulatory system. Then the main organs (heart, liver, spleen, lung, kidney and brain) were collected to estimate the distribution of drug fluorescence. The isolated organ images were captured using a small animal *in vivo* fluorescence imaging system (Carestream, NY, USA).

Determination of PD levels in blood and main organs: The pharmacokinetics of nanodrug was studied by determining the PD concentration of the orbital blood at different time points after tail vein injection of PD-MC (dose: 2.5 mg/kg for PD). In addition, the PD contents in main organs were also quantified at 36 h post-injection of PD-MC. The obtained blood samples or isolated organs (Heart, Liver, Spleen, Lung, and kindney) were weighed and homogenized in 1 mL of acetate buffer (pH~5.0). Then, 0.5 mL of acetonitrile was added for a second round homogenization. After adding 2 mL of ethyl acetate, PD was extracted from the homogenate by centrifugation for 5 min at 4000 rpm. The supernatant was collected and dried up by nitrogen bubbling. The residue was dissolved in methanol and analyzed using High

Performance Liquid Chromatography (HPLC, Shimadzu LC-20A, Japan). For the HPLC measurement, a mixture of acetonitrile/water (60/40 volume ratio) was used as an eluent at a flow rate of 1.0 mL/min. The PD level was expressed as the percentage of the injected dose (% ID) or normalized to the tissue weight in grams (% ID/g). All plasma pharmacokinetic parameters were analyzed by DAS software (version 2.0, Mathematical Pharmacology Professional Committee of China) using a two compartment model. Formula for pharmacokinetics data: $\% \text{ ID} = C_{blood} \times V_{blood} \times (C_{injected} \times V_{injected})^{-1} \times 100\%$. Formula for bio-distribution data: $\% \text{ ID g}^{-1} = C_{organ} \times V_{organ} \times (C_{injected} \times V_{injected} \times M_{organ})^{-1} \times 100\%$.

Histologic and immunochemical analyses: H&E and Sirius red staining were conducted to respectively evaluate the hepatic morphology and collagen deposition. The tissues of major organs (heart, liver, spleen, lung and kidney) were fixed in 10% neutral buffered formalin, embedded in paraffin and made into 4 μm sections. Then the liver sections were deparaffinized, hydrated and stained following standard methods. For immunochemistry staining, liver sections were deparaffinized, rehydrated, and incubated with 3% hydrogen peroxide in order to block endogenous peroxidase. After heated in 10 mM sodium citrate buffer to retrieve antigen and blocked by 5% BSA solution, the liver sections were sequentially incubated with primary antibodies (CD68, TLR4 and α-SMA) and secondary antibodies. After counterstained with hematoxylin, the liver sections were mounted with neutral balsam medium. For immunofluorescent staining, the liver tissues were fixed in 10% PBS-buffered formalin overnight, sequentially exposed to 10% and 30% sucrose in PBS for 10 h each and finally embedded in Tissue Tek OTC compound (Sakura Finetek, Torrance, CA). After permeabilized by 0.25% Triton X-100, the liver

sections were sequentially incubated with primary antibodies (Cleaved-caspase3, NF-κB p-p65, 4-HNE and NOX-4) and corresponding Alexa Fluor 488 or 594-conjugated secondary antibodies. Additionally, the *in vitro* immunofluorescent analyses for RAW (NF-κB p65) and LX-2 (α-SMA) cells were performed in a similar way as described above. Finally, the areas of interest were captured by an Olympus BX51 microscope (Olympus Co., Tokyo, Japan).

Liver and kidney functional assay: Serum levels of alanine aminotransferase (ALT), aspartate aminotransferase (AST), creatinine (Cr) and urea were measured using standard enzymatic procedures following the manufacturer's instruction.

Hydroxyproline assay: Hepatic hydroxyproline content was assessed using commercial hydroxyproline assay kit following the manufacturer's instructions. Briefly, the liver tissues were hydrolyzed at 95 °C for 25 min, adjusted to pH 6.5 and then filtered through activated charcoal. After centrifugation, the supernatant was collected, mixed with detecting liquid and incubated at 60 °C for 20 min. Finally, the samples were measured by a microplate reader at 550 nm.

Quantitative reverse-transcription (qRT)-PCR and Western blot assays: For qRT-PCR assays, the total RNA of cells or hepatic tissues were extracted using TRIzol reagent (Invitrogen, Carlsbad, CA, USA). RNA was reverse-transcribed using the first-strand cDNA kit (Takara, Dalian, China) and conducted with qRT-PCR using the PrimeScript RT-PCR Kit (Takara, Dalian, China) according to the manufacturer's instructions on a StepOne Plus Real-time PCR System (Applied Biosystems, Foster City, CA, USA). The PCR reaction conditions were set at: 95 °C for 30 s; and 40 cycles of 95 °C/5 s, 60 °C/34 s. For Western blot assays, the total proteins of cells or

hepatic tissues were extracted using ProteoJETTM Mammalian Cell Lysis Reagent (Fermentas, Canada) with phenylmethanesulfonyl fluoride (PMSF). Equal amounts of total proteins were firstly fractionated by SDS-PAGE electrophoresis and transferred onto PVDF membrane (Millipore Corporation, MA, USA). After blocked with 5% nonfat milk in Tris-buffered saline with Tween-20 for 1 h at room temperature, the membranes were incubated with the primary antibodies of interest overnight at 4 °C. After incubated with the corresponding secondary antibodies, the bands were revealed by enhanced chemiluminescence detection reagents (Bio-Rad, USA) and recorded by chemiluminescence system (Clinx, Shanghai, China). Quantification was performed using ImageJ software (National Institutes of Health, USA).

Enzyme-linked immunosorbent assay (ELISA): The cellular and hepatic levels of IL-1β, IL-6 and TNF-α were evaluated by commercial ELISA kits following the manufacturer's instructions. Briefly, the standard substance and samples were added into 96-well plate and incubated for 2 h at 37 °C. Then the supernatant was discarded, replaced by 100μL of biotin-antibody and incubated for 1 h at 37 °C. After three times wash with fresh PBS, 100μL of HRP-avidin was added and incubated for 1 h at 37 °C, followed by incubation with 90μL of TMB substrate. After incubated for 30 min at 37 °C, each well was added wtih 50μL of stop solution, at last the optical density was measured by a microplate reader at 450 nm.

Oxidative stress measurements: HSCs were treated as described in the "Cell Treatment" section and incubated with the redox-sensitive dye, 10 μM dichlorofluorescin diacetate (DCFH-DA) for 30 min. Immediately, the DCFH-DA

fluorescence was observed by Olympus IX71 microscope (Olympus Co., Tokyo, Japan) and also quantitatively measured using a multiwall fluorescence scanner (BioTek, Vermont, USA) at 488 nm. *In vivo*, we investigated the thiobarbituric acid reactive substances (TBARS) for quantitative analysis of hepatic lipid peroxidation, a significant marker of oxidative stress. Briefly, the liver tissues were homogenized by BHT-contained PBS and mixed with SDS lysis solution and 0.52 % (pH 3.5) aqueous solution of thiobarbituric acid. Then the samples were incubated at 95 °C for 40 min, followed by a centrifugation at 3500 rpm for 10 min. Finally, the supernatant was measured by a microplate reader at 532 nm.

Hepatocyte apoptosis analyses: The human hepatocyte cell line, LO2 cells were treated as described in the "Cell Treatment" section. The *in vitro* TUNEL apoptosis detection, Caspase3 activity analysis and Annexin-V/PI apoptosis detection were performed by commercial assay kits according to the manufacturer's instructions. *In vivo*, the liver sections and tissues were respectively used for TUNEL apoptosis detection and Caspase3 activity analysis using commercial assay kits (Beyotime, Beijing, China) following the manufacturer's instructions.

Gene overexpression by the transfection of plasmids: Transient cDNA expression was performed in RAW and LX-2 cells using Lipofectamine 3000 reagent according to the manufacturer's protocol. An expression plasmid containing the TLR4 or NOX4 gene was used and the overexpression of TLR4 and NOX4 was confirmed by Western blot assays.

Evaluation of Nitric oxide (NO) availability: The liver sinusoidal endothelial cells

(LSECs) were isolated from the C57BL/6 mice induced by CCl_4 for 6 weeks according to the method of a previous report [3]. The LSECs isolated from fibrotic mice were treated as follows: (1) serum-free DMEM, (2) serum-free DMEM with 10 μM polydatin, (3) serum-free DMEM with polydatin-loaded nano-micelle (PD-MC, loaded with 10 μM polydatin), (4) serum-free DMEM with blank nano-micelle (MC, without polydatin). After 24 h, the NO levels in LSECs were assessed by the DAF-FM-DA probe (Beyotime Biotechnology, Shanghai, China) according to the manufacturer's protocol. For the *in vivo* study, the cyclic guanosine monophosphate (cGMP), a marker of NO availability, of the fibrotic liver tissues was measured using an enzyme immunoassay (Cayman Chemical, Ann Arbor, MI, USA) according to the manufacturer's protocol.

Measurement of TXB_2: The LSECs isolated from fibrotic mice were treated as described above. After 24 h, arachidonic acid (40 μM) was added for 30 min in order to stimulate the prostanoid production of LSECs. Next, the supernatant was collected and the TXA_2 (using its stable metabolites TXB_2) was analyzed by an enzyme-linked immunosorbent assay kit (Cayman Chemical, Ann Arbor, MI, USA) according to the manufacturer's instructions. For the *in vivo* study, a flow-controlled perfusion system was conducted in fibrotic mice as previously described [4]. The perfusate was collected and quantified for the analysis of TXB_2.

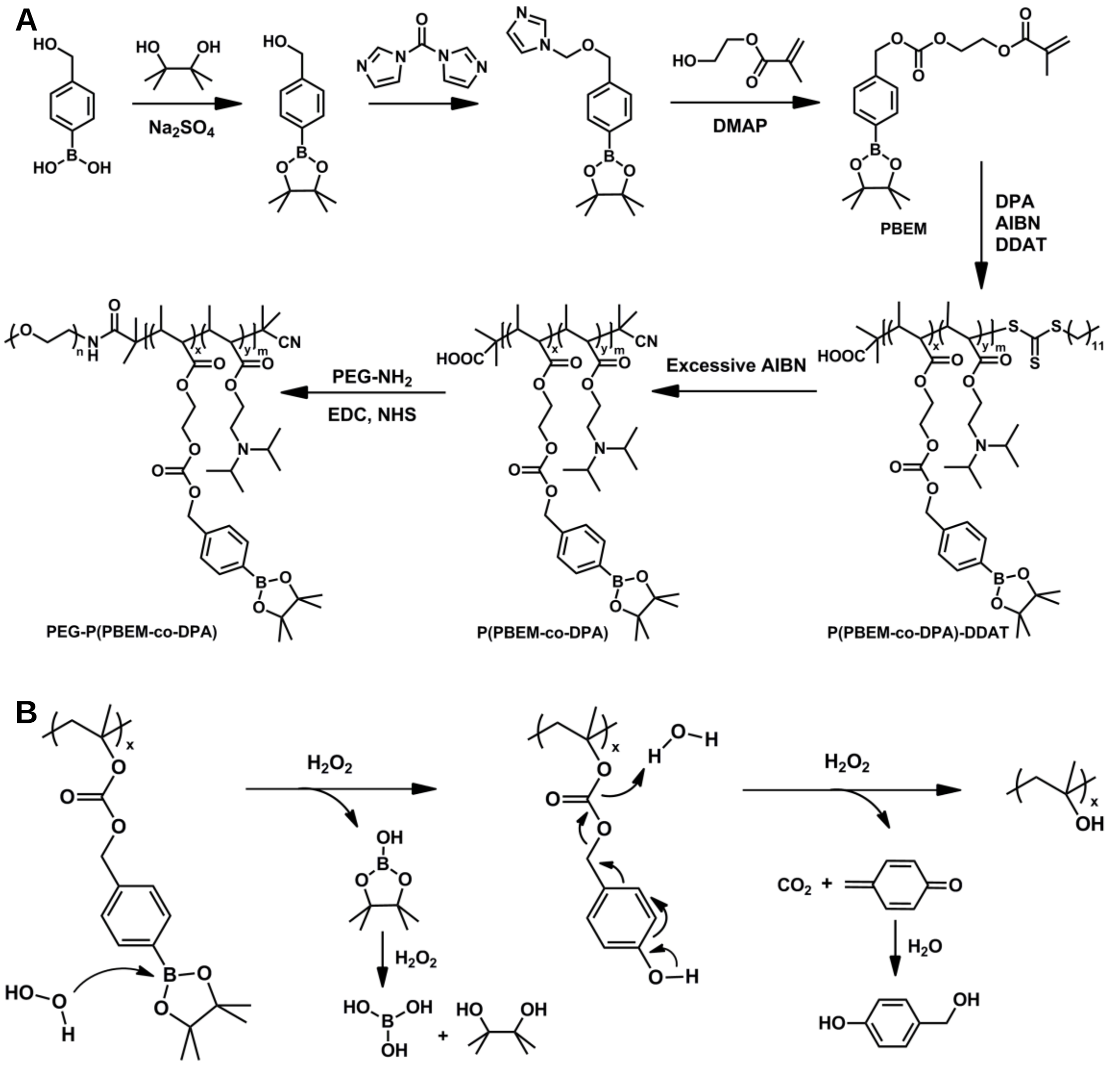

Scheme S1. (A) Synthetic routes for the preparation of PEG-P(PBEM-co-DPA). (B) H_2O_2-responsive oxidation and hydrolysis routes of PBEM.

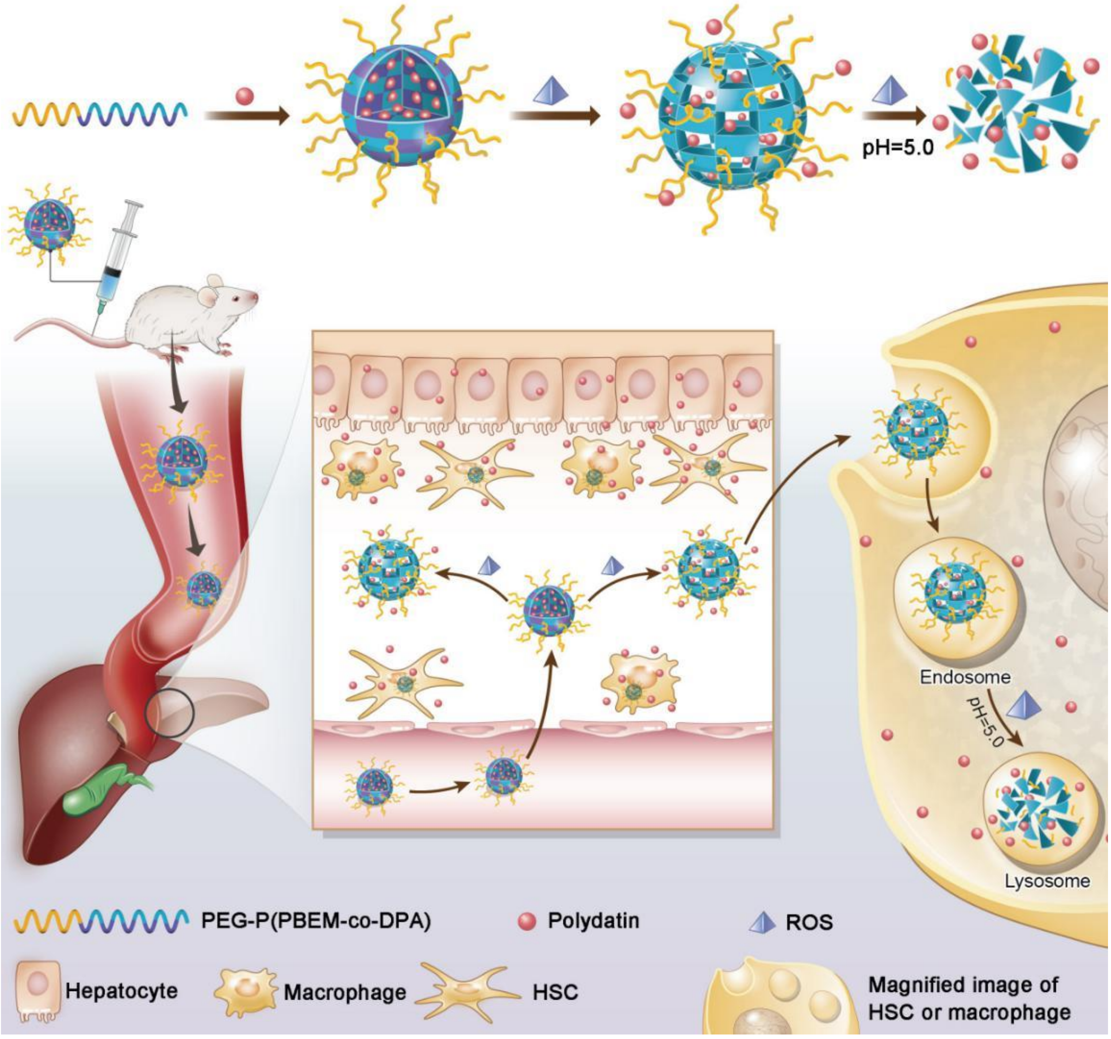

Scheme S2. Schematic illustration of PEG-P(PBEM-co-DPA)-Polydatin as a ROS and pH dual-responsive nanodrug to regulate multiple cell types for the treatment of liver fibrosis.

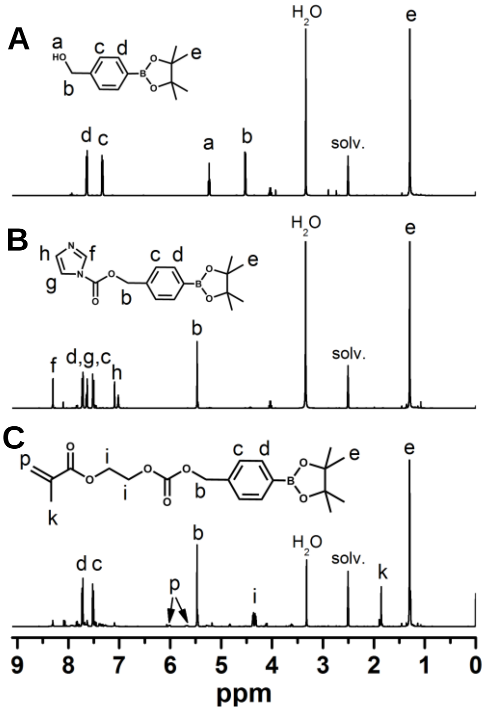

Figure S1. ^{1}H-NMR spectra of 4-(hydroxymethyl)phenylboronic acid pinacol ester (A), CDI-activated pinacol boronic ester (B), and PBEM monomer (C).

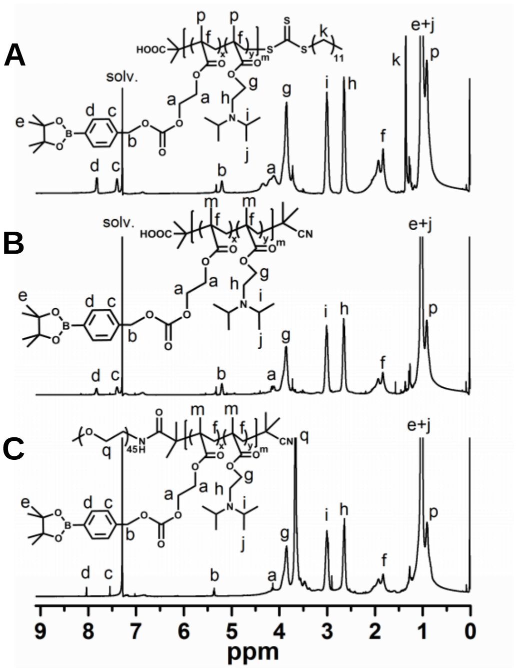

Figure S2. The ^{1}H-NMR spectra of P(PBEM-co-DPA)-DDMAT (A), P(PBEM-co-DPA) (B), and PEG-P(PBEM-co-DPA).

Table S1. Molecular weight and polydispersity of the copolymers

Sample name	Mn [a] (Da)	Mn [b] (Da)	PDI
P(PBEM-co-DPA)	7400	9100	1.14
PEG-P(PBEM-co-DPA)	9400	11200	1.20

[a] Calculated based on 1H NMR spectra. [b] Determined by GPC analyses.

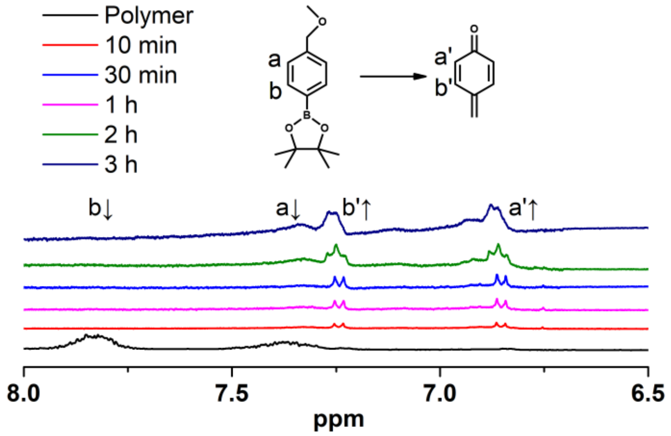

Figure S3. H_2O_2-responsive oxidation and hydrolysis of PEG-P(PBEM-co-DPA) under 0.1 mM H_2O_2 detected by ^{1}H-NMR spectroscopy.

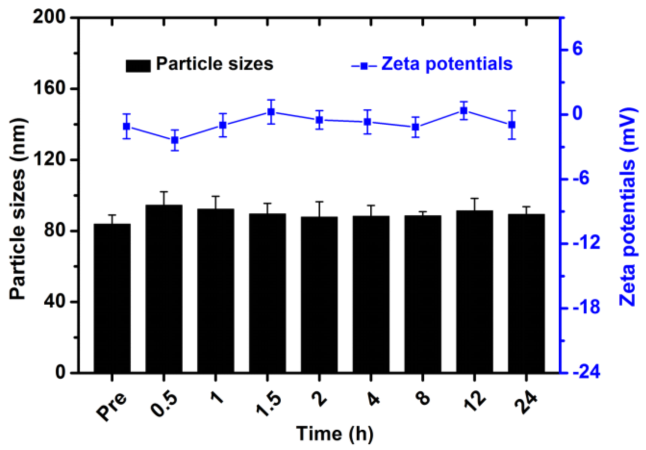

Figure S4. Stability of PD-MC in PBS containing 10% fetal bovine serum (FBS) measured by dynamic light scattering (DLS). Data are mean ± SD. n = 3.

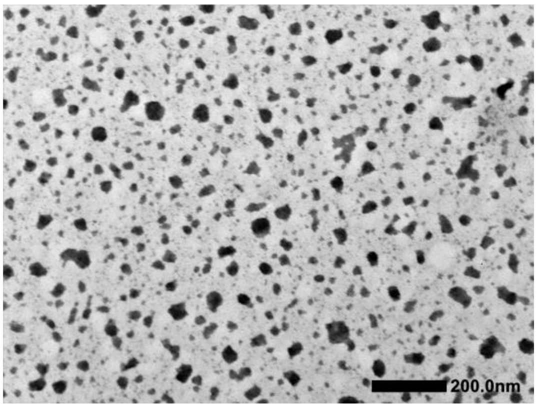

Figure S5. Transmission electron microscopy (TEM) images of PD-MC at pH 5.0.

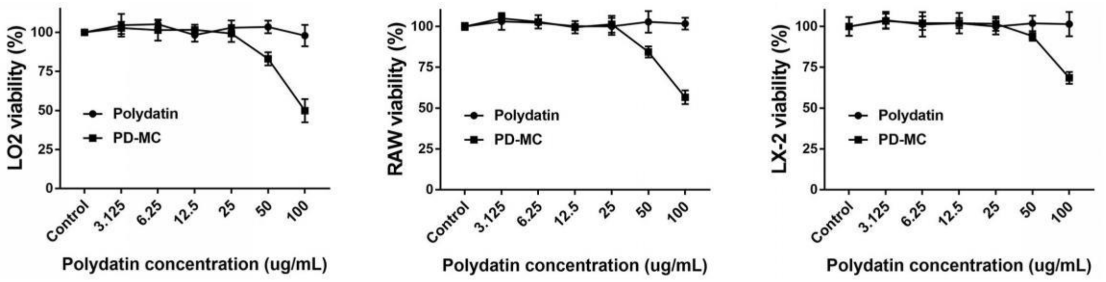

Figure S6. The cytotoxicity of polydatin and polydatin loaded nanomicelle in LO2, RAW and LX-2 cells at different concentrations of polydatin.

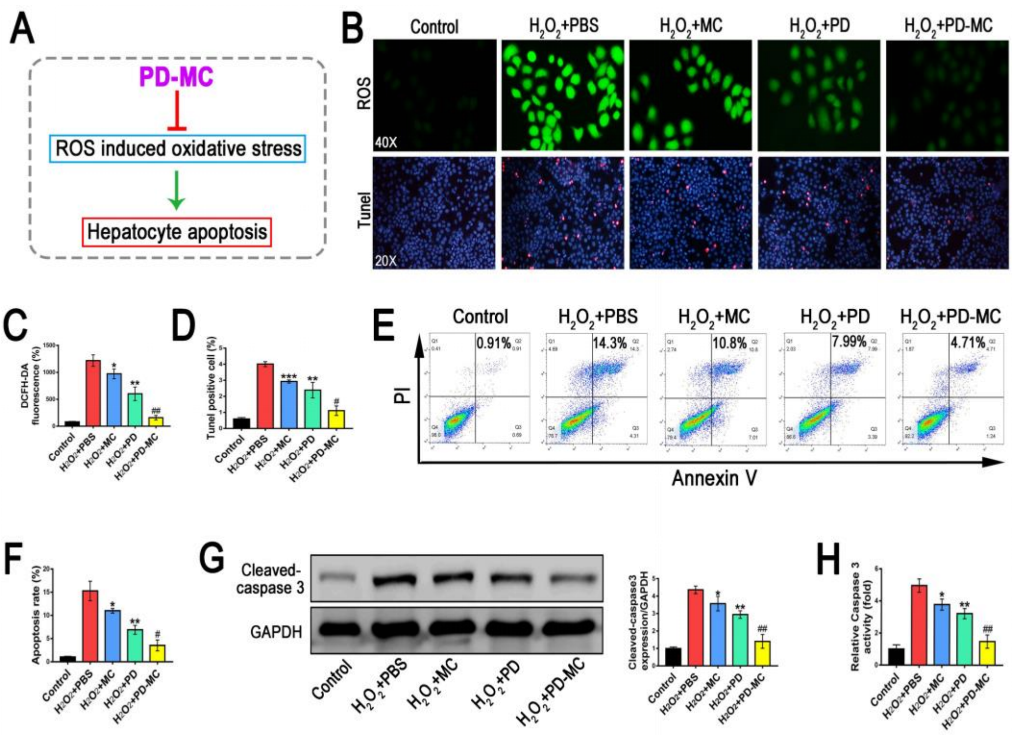

Figure S7. Polydatin-loaded micelle (PD-MC) attenuated H_2O_2 induced apoptosis in LO2 cells. (A) Schematic illustration of the anti-apoptosis mechanism of PD-MC in hepatocytes. (B) & (C) & (D) The levels of oxidative stress and apoptosis were measured by DCFH-DA probe and TUNEL assay, respectively. (E) & (F) Quantitative analysis of apoptotic cells by Annexin V/PI flow cytometry assay. (G) The protein expression of Cleaved-caspase 3 was examined by Western blot assay. (H) Caspase 3 activity of H_2O_2 stimulated LO2 cells with different treatments. n = 3. *$p < 0.05$, **$p < 0.01$, and ***$p < 0.001$ versus LO2 induced by H_2O_2. #$p < 0.05$, ##$p < 0.01$ versus H_2O_2 induced LO2 cells treated with free polydatin.

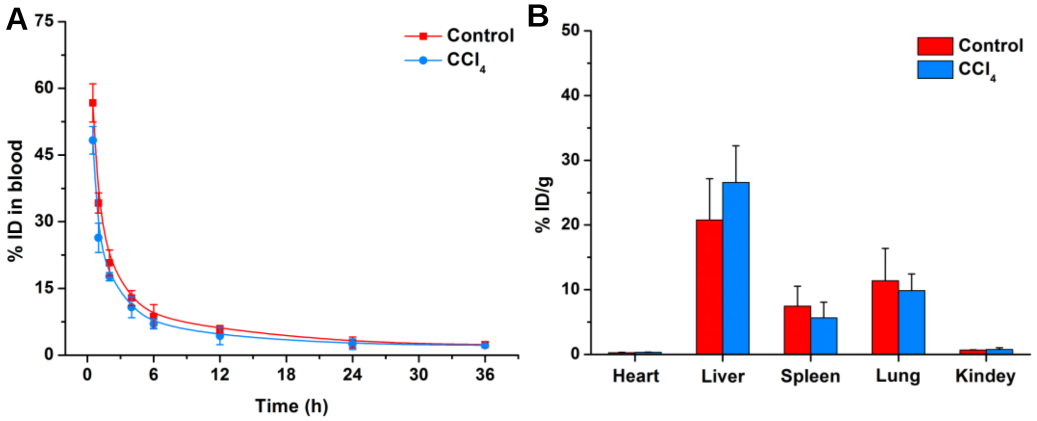

Figure S8. (A) Pharmacokinetic curves of PD-MC according to the PD contents in bloodstream determined by HPLC, shown as the percentage of injected dose (% ID) [5]. Elimination half-lives ($t_{1/2,\ elim}$) were calculated to be 11.04 h and 9.37 h for the control group and CCl_4 treatment group, respectively. n = 3. (B) Quantitative analysis of PD distribution in major organs at 36 h after injection of PD-MC (dose: 2.5 mg/kg for PD). n = 3.

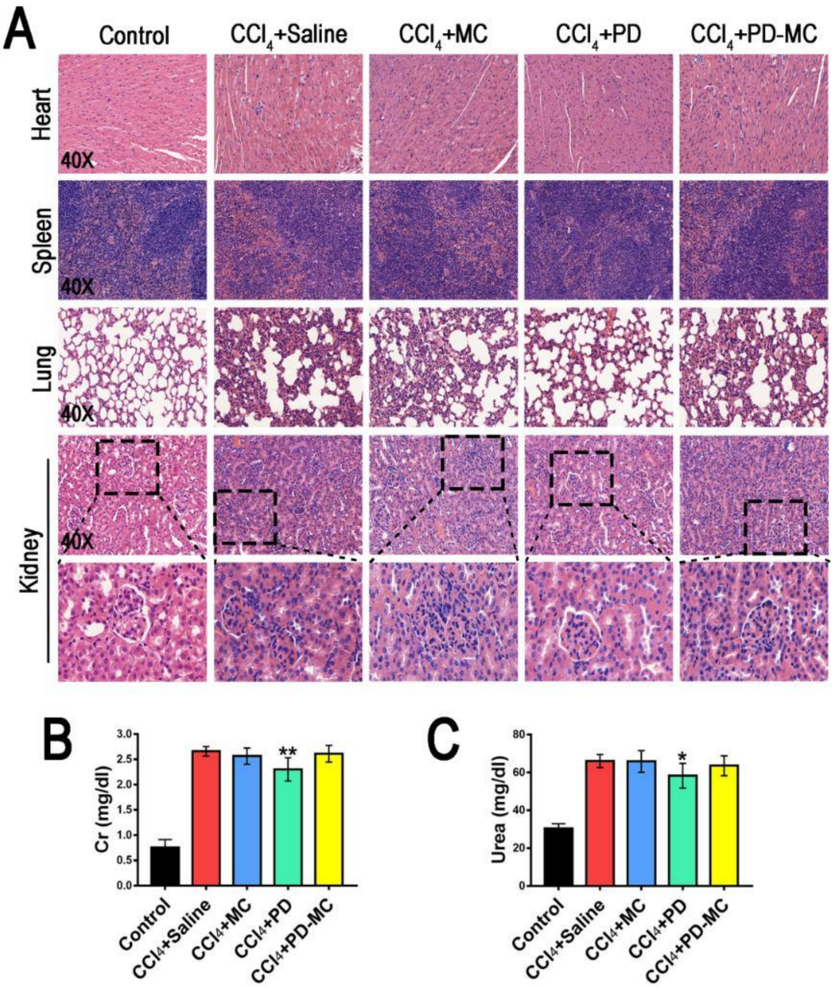

Figure S9. Evaluation of side effects in mice. (A) Tissue inflammation and structural damage examined by pathological H&E staining of the heart, spleen, lung, and kidney. (B) & (C) Analyses of serum function markers of the kidney (Creatinine and Urea). n = 6. Cr: Creatinine, *$p < 0.05$ and **$p < 0.01$ versus mice induced by CCl_4.

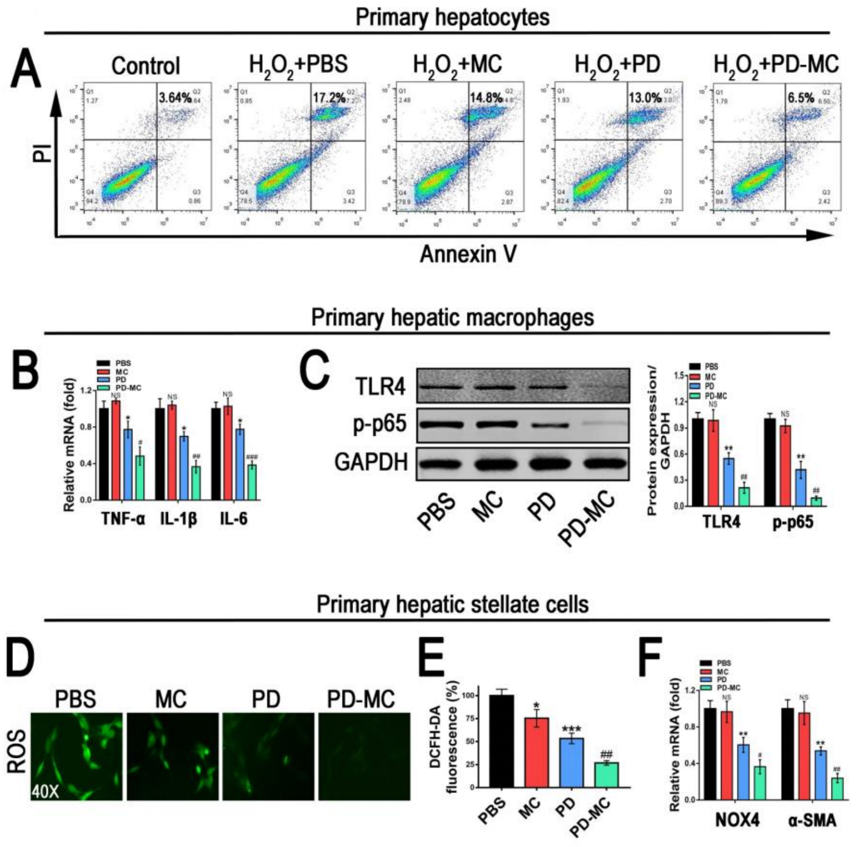

Figure S10. PD-MC reduced hepatocyte apoptosis and averted the activation of macrophages and hepatic stellate cells (HSCs). (A) The apoptosis levels of primary hepatocytes isolated from the CCl_4 induced mice were measured by Annexin V/PI flow cytometry assay. (B) The mRNA levels of TNF-α, IL-1β and IL-6 in primary hepatic macrophages isolated from the CCl_4 induced mice were measured by qRT-PCR. n = 3. (C) The protein levels of TLR4/NF-κB p65 signaling pathway in primary hepatic macrophages were measured by Western blot assay. n = 3. (D) & (E) The levels of oxidative stress in primary HSCs isolated from the CCl_4 induced mice were detected by DCFH-DA probe. n = 3. (F) The mRNA levels of NOX4 and α-SMA in primary HSCs were measured by qRT-PCR. n = 3. NS: no significance, *p < 0.05, **p < 0.01, and ***p < 0.001 versus cells treated with PBS. #p < 0.05, ##p < 0.01, and ###p < 0.001 versus cells treated with free polydatin.

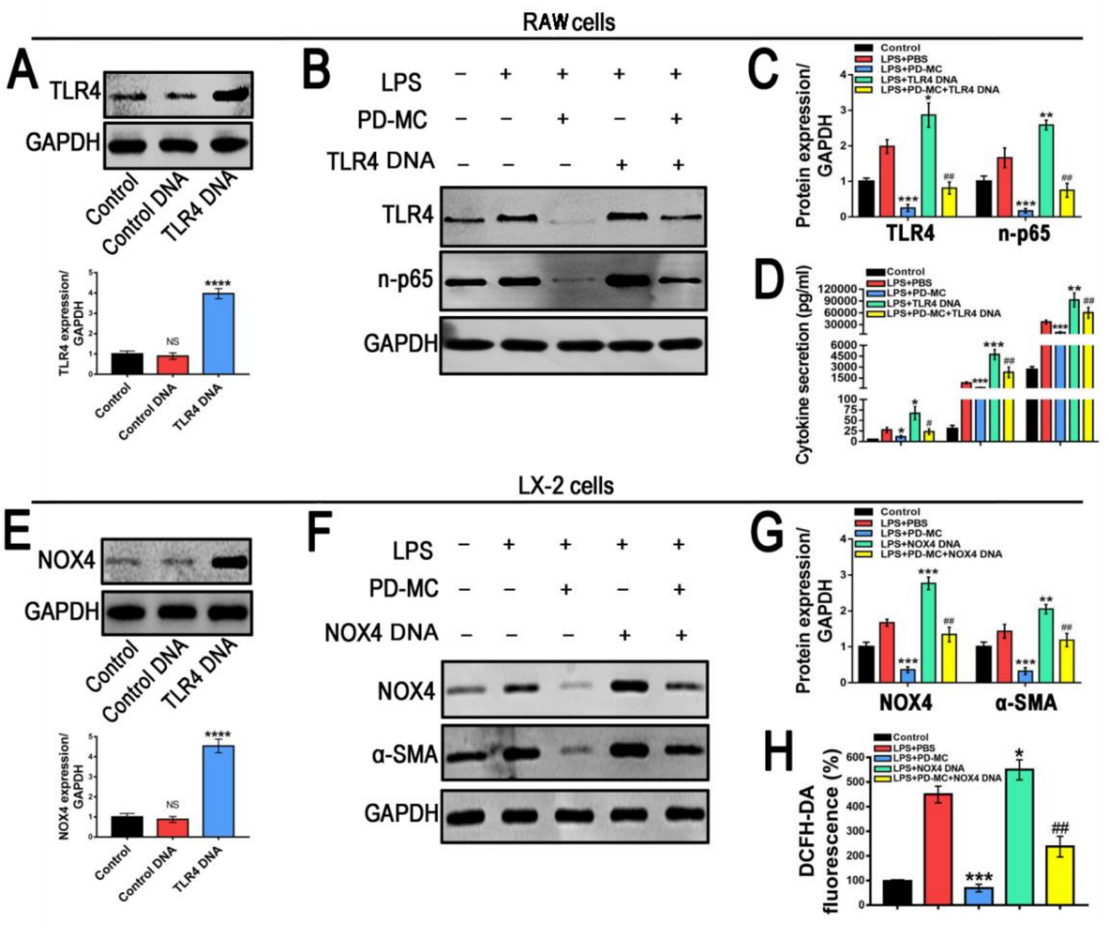

Figure S11. TLR4 and NOX4 were implicated in the effect of PD-MC. (A) The TLR4 overexpression by plasmid DNA transfection was verified by Western blot assay. *n* = 3. NS: no significance, ****p < 0.0001 versus control cells. (B) & (C) Effects of PD-MC on the protein levels of TLR4 and nuclear NF-κB p65 in the LPS induced RAW cells with or without TLR4 overexpression. *n* = 3. n-p65: nuclear NF-κ B p65. *p < 0.05, **p < 0.01, and ***p < 0.001 versus cells treated with LPS. ##p < 0.01 versus cells treated with LPS + PD-MC. (D) The secretion levels of TNF-α, IL-1β and IL-6 were measured by ELISA. *n* = 3. *p < 0.05, **p < 0.01, and ***p < 0.001 versus cells treated with LPS. #p < 0.05 and ##p < 0.01 versus cells treated with LPS + PD-MC. (E) The NOX4 overexpression by plasmid DNA transfection was verified by Western blot assay. *n* = 3. NS: no significance, ****p < 0.0001 versus

control cells. (F) & (G) Effects of PD-MC on the protein levels of NOX4 and α-SMA in the LPS induced LX-2 cells with or without NOX-4 overexpression. $n = 3$. **p < 0.01, and ***p < 0.001 versus cells treated with LPS. ##p < 0.01 versus cells treated with LPS + PD-MC. (H) The levels of oxidative stress in LX-2 cells were measured by DCFH-DA immunofluorescence assay. $n = 3$. *p < 0.05, and ***p < 0.001 versus cells treated with LPS. ##p < 0.01 versus cells treated with LPS + PD-MC.

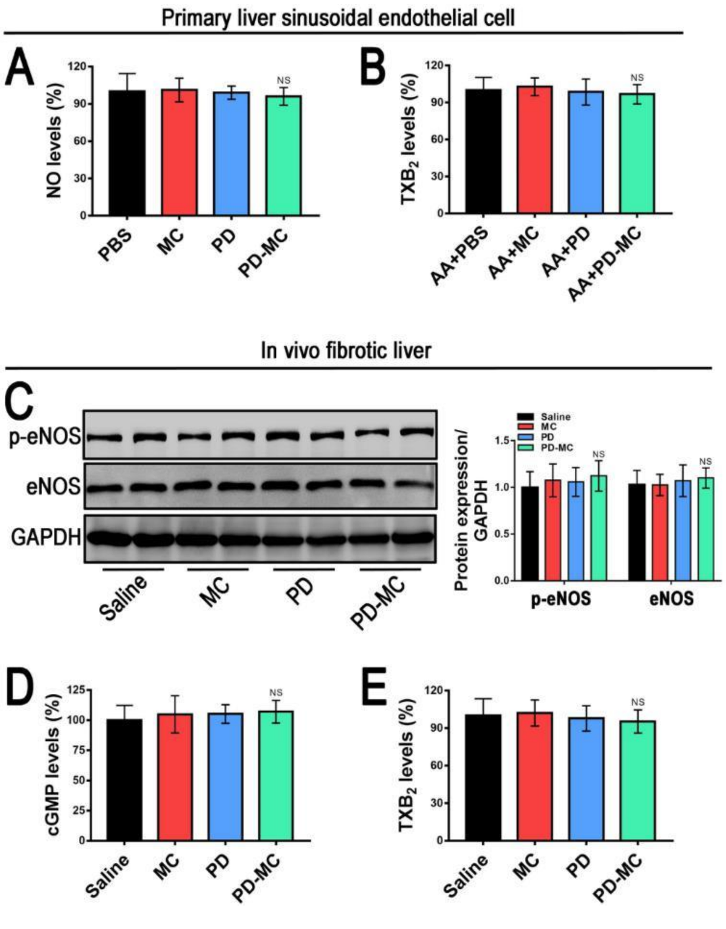

Figure S12. PD-MC exerted no effect on the nitric oxide (NO) availability and thromboxane A_2 (TXA_2) production in the liver sinusoidal endothelial cells (LSECs) and the fibrotic liver. (A) DAF-FM-DA fluorescent detection of intracellular NO in fibrotic LSECs isolated from CCl_4 induced mice. $n = 3$. NS: no

significance versus cells treated with PBS. (B) Relative TXB_2 (stable metabolites of TXA_2) production in fibrotic LSECs induced by arachidonic acid (AA). n = 3. NS: no significance versus cells treated with PBS. (C) Hepatic eNOS activation was determined by Western blot assay of the phosphorylated eNOS/total eNOS ratio in CCl_4 induced mice receiving different treatments. n = 4 (D) Hepatic cGMP levels (a marker of NO availability) in CCl_4 induced mice receiving different treatments. n = 6. NS: no significance versus fibrotic mice treated with saline. (E) Hepatic TXB_2 production in CCl_4 induced mice receiving different treatments. n = 6. NS: no significance versus fibrotic mice treated with saline.

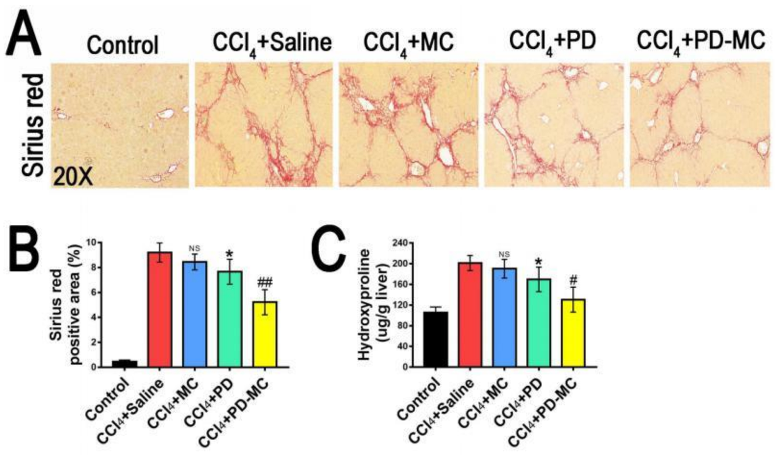

Figure S13. Anti-fibrotic effect of PD-MC in the mice that have already developed liver fibrosis after 6 weeks of CCl_4 treatment. (A) & (B) Representative hepatic histology of Sirius red staining. (C) Hepatic collagen accumulation was evaluated by hydroxyproline content assay. n = 6. NS: no significance and $*p < 0.05$ versus mice treated with CCl_4 + Saline. $\#p < 0.05$ and $\#\#p < 0.01$ versus CCl_4 induced mice treated with free polydatin.

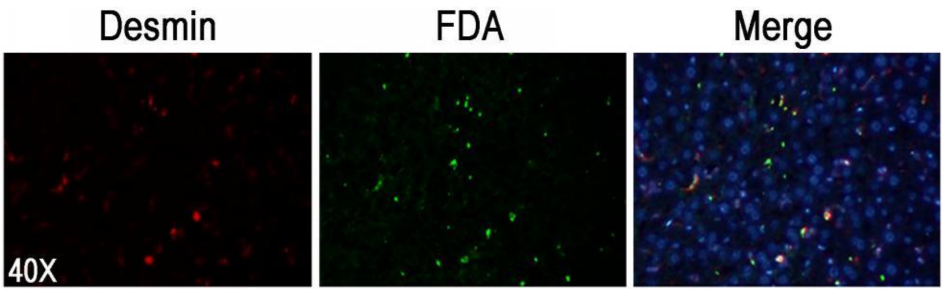

Figure S14. *In vivo* HSC distribution of the nanomicelle. Intrahepatic HSC distribution of FDA-loaded nanomicelle in control healthy mice at 24 h after i.v. injection of FDA-loaded nanomicelle. The locations of HSCs were indicated by the immunofluorescent staining of Desmin.

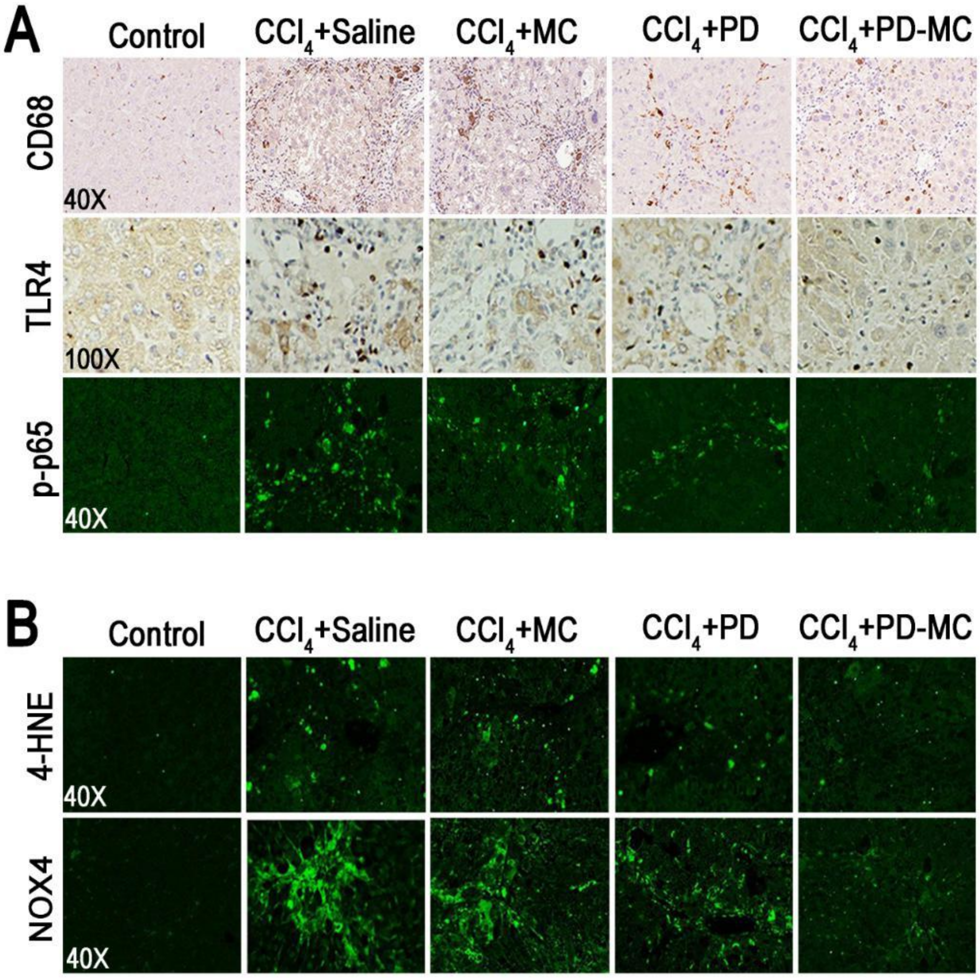

Figure S15. Increased magnification of the images in Figure 7.

References

(1) Chen, W.; Yuan, Y.; Cheng, D.; Chen, J.; Wang, L.; Shuai, X. *Small* **2014,** 10(13), 2678-87.

(2) Goth, L. *Clin. Chim. Acta.* **1991,** 196(2), 143-151.

(3) Paik, Y.H.; Iwaisako, K.; Seki, E.; Inokuchi, S.; Schnabl, B.; Osterreicher, C.H. *Hepatology* **2011**, 53, 1730-41.

(4) Le Moine, O.; Louis, H.; Stordeur, P.; Collet, J.M.; Goldman, M.; Devière, J. *Gastroenterology* **1997**, 113, 1701-6.

(5) Han, X.; Li, Y.; Xu, Y.; Zhao, X.; Zhang, Y.; Yang, X.; Wang, Y.; Zhao, R.; Anderson, G.J.; Zhao, Y.; Nie, G. *Nature Communications* **2018,** 9(1).
